# Short-chain fatty acid-producing microbes differentiate non-infectious and infectious neutropenic fever in leukemia

**DOI:** 10.1128/msystems.01343-25

**Published:** 2026-03-31

**Authors:** Samantha Franklin, Pranoti Sahasrabhojane, Tomo Hayase, Eiko Hayase, Chia-Chi Chang, Jayastu Senapati, Sai Prasad Desikan, Tapan Kadia, Philip L. Lorenzi, Robert R. Jenq, Samuel A. Shelburne, Jessica Galloway-Peña

**Affiliations:** 1 Department of Veterinary Pathobiology, Texas A&M University 14736, College Station, Texas, USA; 2 Interdisciplinary Graduate Program in Genetics and Genomics, Texas A&M University 14736, College Station, Texas, USA; 3 Department of Infectious Disease, Infection Control and Employee Health, The University of Texas MD Anderson Cancer Center 4002, Houston, Texas, USA; 4 Department of Genomic Medicine, The University of Texas MD Anderson Cancer Center 4002, Houston, Texas, USA; 5 Department of Leukemia, The University of Texas MD Anderson Cancer Center 4002, Houston, Texas, USA; 6 Department of Bioinformatics and Computational Biology, The University of Texas MD Anderson Cancer Center 4002, Houston, Texas, USA; 7 Department of Stem Cell Transplantation and Cellular Therapy, The University of Texas MD Anderson Cancer Center 4002, Houston, Texas, USA; Drexel University, Philadelphia, Pennsylvania, USA

**Keywords:** microbiome, metabolome, neutropenic fever, bacteremia, machine learning, network analysis

## Abstract

Neutropenic fever (NF) is often the first sign of infection in patients with hematologic malignancies, but its cause is frequently unknown, leading to broad-spectrum antibiotic use without confirmed infections. Although research links gut microbiome disruptions to treatment-related infections, it typically examines NF as the outcome, leaving a gap in understanding how the microbiome and metabolic factors distinguish infectious from non-infectious cases. Stool samples from acute myeloid leukemia patients were analyzed to characterize gut microbiome composition and fecal metabolites at baseline and at fever onset. Machine learning models, network analyses, and functional profiling were used to differentiate infectious NF vs non-infectious NF at baseline and at fever onset. The baseline model (area under the receiver operating characteristic [AUROC] = 0.769) identified higher levels of *Akkermansia*, *Enterobacter*, *Escherichia*–*Shigella*, and *Flavonifractor* as predictors of infectious NF, while *Collinsella*, *Lachnospiraceae*, *Coprococcus*, and acetic acid were linked to non-infectious cases. At fever onset, *Enterococcus* was enriched in infectious NF, whereas *Gemella*, Butyrate, *Lachnospiraceae*, *Ruminococcaceae*, and *Eisenbergiella* abundances favored non-infectious NF outcomes (AUROC = 0.752). Network analyses also revealed greater functional diversity and microbiome–metabolome connectivity in non-infectious cases at fever onset. This study suggests that gut microbiota and metabolites may serve as biomarkers for distinguishing infectious from non-infectious neutropenic fever, warranting further validation in larger cohorts.

**IMPORTANCE:**

Our study tackles the challenge of managing neutropenic fever (NF) in immunocompromised patients whose numbers have increased due to various immunodeficiencies and treatments that suppress immune function. Fever is often the only sign of a serious infection in these patients, yet there are neither clear patterns linking risk factors to infection nor biomarkers reliable for ruling out non-infectious causes. As a result, febrile patients are typically empirically treated for major pathogens, even in the absence of confirmed infections, which propagates antimicrobial resistance and gut dysbiosis. Our research utilizes gut microbiome and targeted metabolomic profiling from two cohorts of patients with acute myeloid leukemia undergoing chemotherapy and employs a machine learning framework to distinguish between infectious and non-infectious NFs at baseline and upon fever onset.

## INTRODUCTION

Neutropenic fever (NF) is often the earliest and most common sign of infection in patients with hematologic malignancies, affecting nearly 80% of those with acute myeloid leukemia (AML) (1). However, in most cases, the underlying cause of fever remains unidentified, and an infectious source cannot be confirmed (2). Despite this uncertainty, current clinical guidelines mandate the immediate use of broad-spectrum antibiotics, even when the likelihood of infection is low (3). While this approach attempts to prioritize patient safety, it highlights significant gaps in our understanding of NF pathogenesis and limits the development of complementary strategies for its prevention and management. Moreover, the widespread use of antibiotics has contributed to rising rates of antimicrobial resistance and *Clostridioides difficile* infections, presenting additional challenges in patient care (4). Additionally, evidence suggests that prophylactic antibiotic use not only may fail to prevent NF but also could prolong its duration or even contribute to its onset in hematologic patients, further underscoring the need for improved management strategies and a better understanding of its etiology (5).

Extensive antibiotic use during chemotherapy can also severely disrupt the gut microbiome, leading to community domination, dysbiosis, and pathogen overgrowth (6–8). This disruption not only may impair the metabolism and absorption of chemotherapeutic agents, potentially increasing toxicity while reducing efficacy but also may contribute to systemic inflammation (9, 10). Chemotherapy-induced cell lysis, drug interactions, and the breakdown of malignant cells often result in sterile inflammation of the intestinal epithelium, driven by damage-associated molecular patterns, as opposed to inflammation and fever caused by microbial pathogen-associated molecular patterns (9, 11). Distinguishing between fever caused by true infections and fever triggered by sterile inflammation due to epithelial tissue damage is critical for improving antibiotic stewardship.

Several observational studies have described the gut microbiome and metabolome in AML patients with NF, revealing significant dysbiosis during the initial occurrence of NF and a loss of microbiota-derived protective metabolites (12, 13). In pediatric hematopoietic stem cell transplant (HSCT) patients, prolonged NF has been linked to more severe gut dysbiosis, while in pediatric lymphoblastic leukemia, microbial biomarkers such as elevated baseline *Faecalibacterium* levels have been associated with a greater infection risk, and increased *Proteobacteria* levels have been correlated with a higher risk of NF (14–16). Additionally, recent research suggests that an expansion of *Akkermansia* in the gut of adult acute leukemia patients may serve as a predictive signature for NF development (17). However, these studies have treated NF as an infectious event, without distinguishing between infectious or non-infectious causes of fever. Thus, this study aims to characterize the gut microbiome and fecal metabolic landscape in AML patients undergoing induction chemotherapy, to improve understanding of how host–microbe interactions contribute to neutropenic fever pathogenesis, and to determine if distinct microbiome and fecal metabolite signatures can differentiate infectious from non-infectious neutropenic fever at baseline and at fever onset.

## MATERIALS AND METHODS

### Study population

Longitudinal fecal samples and 16S V4 rRNA sequences from two cohorts of newly diagnosed adult AML patients undergoing induction chemotherapy (IC) at MD Anderson Cancer Center (MDACC) in Houston, TX, were used in this study. Stool samples were collected from September 2013 to September 2015 for the first cohort (PA13-0339) and from January 2015 to February 2020 for the second cohort (PA15-0780). Aspects of these cohorts and sample collection were previously published, and 16S V4 rRNA sequences were deposited in the NCBI Sequence Read Archive (http://www.ncbi.nlm.nih.gov/sra) under the BioProject IDs PRJNA352060 and PRJNA526551 (PA13-0339) and PRJNA1124986 (PA15-0780) (18–20). Patient cytogenetic abnormalities and baseline AML-associated mutations assessed as part of a comprehensive routine molecular profiling panel for prognosis and disease monitoring at MDACC were retrospectively collected from electronic medical records. Patient demographics, outcome classifications, chemotherapy, antibiotic administration, and treatment responses were also retrospectively collected from electronic medical records (Table S1).

### Clinical definitions

NF was initially classified according to the Infectious Diseases Society of America criteria and categorized into four standard groups based on established clinical trial guidelines: (i) microbiologically defined infection (MDI), (ii) clinically defined infection (CDI), (iii) fever of unknown origin (FUO), and (iv) non-infectious fever (21, 22). For our analysis, we reclassified patients into three groups: infectious NF, non-infectious NF, and no fever. Infectious NF included patients who had either an MDI or a CDI and had a positive blood culture. Non-infectious NF included patients with FUO and NF with no infectious isolate or symptomology. The no-fever group consisted of patients who neither developed a fever nor had any infectious isolate identified.

### Sample collection

Two sampling time points were used in this study: baseline and NF onset (referred to hereafter as onset). Baseline was defined as the stool sample collected closest to the initiation of IC, with an average collection time of 1 day after IC start. Onset was defined as the stool sample collected closest to the onset of NF, using a sampling window of 4 days before to 3 days after fever onset. Samples collected outside of this window were excluded from the study. For patients having an infectious NF, the average time of collection was 1 day after fever onset and, on average, 2 days before the first positive blood culture. For patients who did not develop NF, an onset-equivalent sample collected at the average time to fever onset from the start of chemotherapy (16 days) was included if it was collected within the pre-defined sampling window.

Due to sample availability, the patient cohorts included in the baseline and onset analyses were not identical. Of the total patients analyzed, 49 were represented in both the baseline and onset groups. The remaining (21 baseline-only and 25 onset-only) patients were unique to each time point. This sharing of patients across time points should be considered when interpreting associations across groups, as each analysis may reflect distinct subsets of the study cohort. However, each patient’s baseline and onset samples were obtained at temporally distinct time points, with no overlap or repetition of stool specimens between cohorts (Table S2). Batch and sequencing center were included as covariates in downstream analyses to account for variability associated with differences in sequencing date and sequencing facility.

### Fecal microbiome and metabolomic profiling

DNA was extracted from approximately 50 mg of stool using a bead-beating protocol with mechanical lysis, followed by purification with QIAamp DNA Minikit (Qiagen, catalog no. 51306). The V4 region of the 16S rRNA gene was amplified using the 515F/806R primer pair developed by the Earth Microbiome Project and sequenced on the Illumina MiSeq platform, as previously described (23).

Targeted metabolomics was performed using 100–200 mg of snap-frozen stool. Metabolites were extracted in 1 mL of ice-cold 0.1% ammonium hydroxide in 80:20 methanol:water (vol/vol) and centrifuged at 17,000 × *g* for 5 min at 4°C, and the supernatants were evaporated to dryness under nitrogen. Dried extracts were reconstituted in deionized water, and 10 μL was injected for ion chromatography–mass spectrometry (IC-MS) analysis. Separation was performed using a Thermo Scientific Dionex ICS-5000+ system equipped with an IonPac AS11 column (4 µm, 250 × 2 mm) at 30°C, with a mobile phase gradient of water and 100 mM KOH in water at a flow rate of 360 μL/min over 50 min. Methanol was infused post-column to enhance desolvation and sensitivity. Mass detection was conducted using a Thermo Orbitrap Fusion Tribrid Mass Spectrometer in negative electrospray ionization mode at a resolution of 240,000. Raw data were processed in Thermo TraceFinder 5.1, and metabolite abundances were normalized to sample weight. The 10 targeted fecal metabolites were 2-hydroxyglutarate (2-HG), acetic acid, butyrate, glyceric acid, ketoleucine, lactate, malate, pentanoic acid, propionic acid, and succinate.

### Statistical analysis

After taxonomic classification, features present in <10% of patients or with abundance of <0.1% were filtered out. α-Diversity and relative abundance groups were compared using Mann–Whitney *U*-tests. Categorical variables were analyzed with Fisher’s exact test or *χ*^2^ test. β-Diversity was assessed using principal coordinate analysis with weighted and unweighted UniFrac distances, and group differences were tested using permutational multivariate analysis of variance (PERMANOVA). A post hoc pairwise PERMANOVA test was applied after a significant *P* value was achieved with the global PERMANOVA test (24). Taxonomic names are reported exactly as assigned by the SILVA database (SSURef_NR99_119 database) to preserve consistency and reproducibility of classification. Associations were considered statistically significant at *P* < 0.05 before correction. Multiple testing correction was performed using the Benjamini–Hochberg (BH) method to control the false discovery rate. Both adjusted and unadjusted *P* values are reported; however, no results remained significant after adjustment, consistent with the known stringency of the correction in sparse settings. All analyses were performed in R (v.4.3.1). Microbial and metabolite variables with a Pearson correlation of >0.9 were removed to reduce potential multicollinearity. Metagenome functional composition was predicted using 16S V4 rRNA sequences with PICRUSt2 (v.2.5.2) (25) and annotated via the MetaCyc database (26) and thus represents predicted, not directly measured, pathway abundances. Differential abundance analysis was performed with *lin*ear regression framework for *d*ifferential *a*bundance analysis (LinDA) with Bonferroni adjusted *P* values (27).

### Model optimization and creation

#### Variable selection

To optimize model performance and interpretability, four variable screening methods were tested before building a selection model: all raw variables (model A); all variables with CLR-transformed microbial variables, where values of 0 were replaced with a pseudocount of the smallest non-0 value present within the data set divided by 2 [min(relative abundance) / 2] (model B); univariate screening using *χ*^2^, Fisher’s exact, and Mann–Whitney tests with a threshold of *P* < 0.3 (model C); and CLR transformation on microbial variables passing univariate selection (model D). Following these screening methods, four variable selection models were created, and their performance was compared.

#### Aggregate model design

Supervised machine learning was used in which an algorithm learns a mapping from input features (microbial taxa, metabolites, and clinical features) to a known outcome (infectious vs non-infectious NF), implemented as an extreme gradient-boosting (XGBoost) tree-based classifier. Patients were randomly divided into training (80%) and test (20%) sets, stratified by outcome. XGBoost was employed for feature selection using the R package “xgboost” (v.1.7.5.1) (28). Each model was trained using fivefold cross-validation with 10 repeats, implemented with a “gbtree” booster. Model performance was evaluated using prediction accuracy and the area under the receiver operating characteristic (AUROC) curve.

To enhance model robustness and minimize clinical data variability, the modeling procedure was repeated on 100 independent stratified random splits of the data (using an 80–20 training–test ratio), and the results were aggregated (29). Variable importance scores were averaged across models, and variables with a mean importance score of ≥0.3 were selected and utilized to create a final model using the same aggregate method described above.

#### Machine learning optimization

Hyperparameter selection followed a “coarse-to-fine” optimization using iterative random searches within progressively refined search spaces until an optimal range was found. Each random search ran for up to 500 iterations, followed by a final grid search on the optimal space. Search spaces were refined from default parameters to maximize test accuracy and AUC. Seven parameters (nrounds, max_depth, min_child_weight, subsample, colsample_bytree, eta, and gamma) were tuned (Table S3). The SHAPforxgboost package (v.0.1.3) computed the *Sh*apley *a*dditive ex*p*lanation (SHAP) values for each feature and generated a summary plot to visualize feature contributions (30).

### Network analysis

Relationships between biomarkers and all other variables were evaluated using Spearman’s rank correlation test, applied to non-transformed relative abundance data. Correlations with an absolute coefficient of ≥0.6 were included in the network analysis. To improve interpretability, we focused on two connection levels: primary, which represents direct correlations between biomarkers and other variables, and secondary, which involves correlations with variables that are directly connected to biomarkers (i.e., second-order adjacency). This approach captured immediate and next-level associations while excluding distant relationships. The network was visualized using Gephi software (v.10.1) (31).

## RESULTS

### Patient characteristics

In the baseline analyses, 70 patients were included: 33 experienced infectious NF; 14 had non-infectious NF; and 23 had no fever (Table S4). For the onset analyses, 74 patients were included, with 45 classified as infectious NF, 15 as non-infectious NF, and 14 with no fever (Table 1). No clinical variables were significantly associated with outcomes in the baseline group. However, in the onset group, chemotherapy intensity (*χ*^2^ *P* = 0.007) and type (*P* = 0.029) were significantly associated with NF outcome, as were the use of a cephalosporin (*P* = 0.002) and carbapenem (*P* = 0.0001) for more than 72 consecutive hours (Fig. S1A through D). These associations reflect differences in the patient populations analyzed at each time point. To further characterize potential selection bias arising from patients contributing samples at more than one time point, we compared key clinical characteristics among patients who contributed samples only at baseline, only at onset, or at both time points. This analysis showed that only prolonged cephalosporin and carbapenem exposure, as well as the incidence of NOTCH1 mutation, differed significantly across these three groups (Table S5). Although this heterogeneity should be considered when interpreting baseline vs onset associations, the statistical differences in antibiotic exposure between those two time points are completely expected.

**TABLE 1:** Characteristics of the patients included in the NF onset analyses^*c*^

Patient characteristics	Infectious NF	Non-Infectious NF	No fever	*P* value^*b*^	Test statistic
Patient count, *N*	45	15	14		
Sex, *N*				0.899	0.213
Female	24	7	7		
Male	21	8	7		
Days to NF, median (range)	16 (0–135)	11 (0–31)			
Days to infection, median (range)	19 (1–135)				
Chemotherapy intensity, *N*				0.007	9.908
High	29	11	3		
Low	16	4	11		
Chemotherapy type, *N*				0.029	14.037
Fludarabine	6	5	1		
Non-fludarabine (high)	23	6	2		
Hypomethylators	13	2	8		
Other (low)	3	2	3		
Antibiotic administration^*a*^, *N*					
Pip-Tazo	14	2	1	0.108	4.455
Cephalosporin	30	10	2	0.002	12.690
Carbapenem	32	7	1	0.0001	18.006
AML somatic mutations, *N*					
TET2	20	6	9	0.352	2.088
RAS	17	5	4	0.809	0.424
DNMT3	12	6	4	0.616	0.968
FLT3-ITD	13	4	3	0.860	0.303
NPM1	11	5	2	0.490	1.428
ASXL1	9	2	5	0.314	2.319
IDH2	11	4	2	0.681	0.768
IDH1	9	1	0	0.110	4.409
CEBPA	4	3	3	0.348	2.114
KIT	4	1	3	0.354	2.077
RUNX1	6	1	1	0.684	0.759
TP53	8	0	0	0.056	5.781
NOTCH1	5	3	0	0.222	3.015
GATA2	6	1	1	0.684	0.759
Complex karyotype, *N*	10	3	1	0.450	1.597

^*a*^ *P* and *χ*^2^ values were determined via *χ*^2^ test using *R*.

^*b*^ Antibiotic administration is defined as being administered for at least 72 consecutive hours.

^*c*^ NF, neutropenic fever.

### Gut microbiome α- and β-diversity differ based on infection status at fever onset but not at baseline

**Fig 1:**
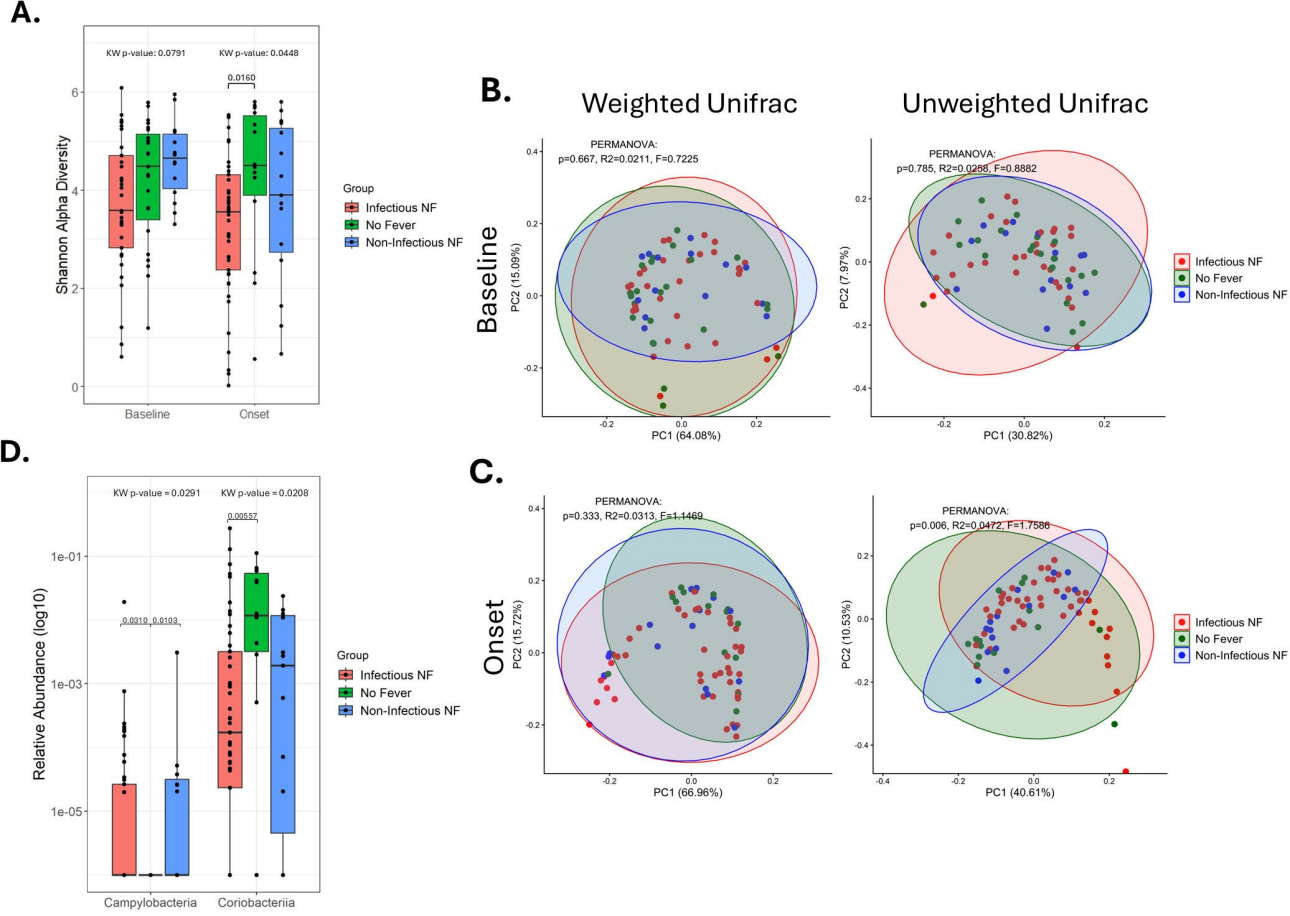
Comparison of the diversity of the gut microbiome across outcome groups. (**A**) The Shannon diversity index was used to analyze the α-diversity across outcome groups. Box and whisker plots extend from the 25th to 75th percentiles, and the whiskers extend to the minimum and maximum values. Dots represent individual values. Kruskal–Wallis tests were used to analyze within time-period α-diversity, and *P* values were reported on the plot. If significant, Dunn’s test for multiple comparisons was performed, and significant *P* values were reported on the plot. β-Diversity was assessed by principal coordinate analysis of weighted and unweighted UniFrac distances at baseline (**B**) and at the onset of NF (**C**). Each point represents a single sample; colors indicate the outcome group. Ellipses were drawn with 95% confidence level. The results of a PERMANOVA test are shown on each plot. (**D**) Box and whisker plot depicting the log10 scaled relative abundance for the named taxa. Abbreviations: NF, neutropenic fever; PERMANOVA, permutational multivariate analysis of variance.

To assess overall gut microbiome differences among infectious NF, non-infectious NF, and no-fever groups, pre-existing 16S rRNA V4 region sequencing data from baseline and onset stool samples were analyzed. Shannon α-diversity was compared at both time points (Fig. 1A). At baseline, no significant difference was found (Kruskal–Wallis *P* = 0.079), though α-diversity trended higher in the non-infectious NF group compared to the other two groups. At onset, α-diversity differed significantly among the three clinical groups (Kruskal–Wallis *P* = 0.045), with Dunn’s test showing a lower diversity in infectious NF compared to no fever (*P* = 0.016). The non-infectious NF group had slightly higher diversity than infectious NF, though this difference was not statistically significant.

No statistical difference in β-diversity was observed between groups at baseline (Fig. 1B). At onset, only unweighted UniFrac distances showed significant differences (*P* = 0.006) in overall community composition among groups (Fig. 1C). Post hoc pairwise PERMANOVA tests, with BH correction, revealed significant differences between the infectious NF and no-fever groups (*P*adj = 0.021), as well as between infectious NF and non-infectious NF groups (*P*adj = 0.021). No significant differences were detected between no-fever and non-infectious NF groups (*P*adj = 0.634), with the non-infectious NF group clustering more tightly (Fig. 1C). Since unweighted UniFrac emphasizes the presence or absence of taxa rather than their relative abundance, this suggests that non-infectious NF patients may share a more similar microbial community with each other compared to the other two groups whose ellipses are wider.

Relative abundances at the class level were compared across all three groups using a Kruskal–Wallis test followed by Dunn’s post hoc test for multiple comparisons. At baseline, no class-level differences reached statistical significance (Fig. S1E; Table S6). At onset, however, significant differences were observed for Campylobacteria (Kruskal–Wallis *P* = 0.029) and Coriobacteriia (*P* = 0.021) across the groups (Fig. 1D; Table S7). Specifically, the no-fever group had lower Campylobacteria abundance compared to both infectious NF (Dunn’s *P* = 0.031) and non-infectious NF (*P* = 0.010) groups, while Coriobacteriia abundance was significantly higher in the no-fever group compared to the infectious NF group (Dunn’s *P* = 0.006).

### Bacterial composition and functional profiles differ among fever outcome groups at baseline

**Fig 2:**
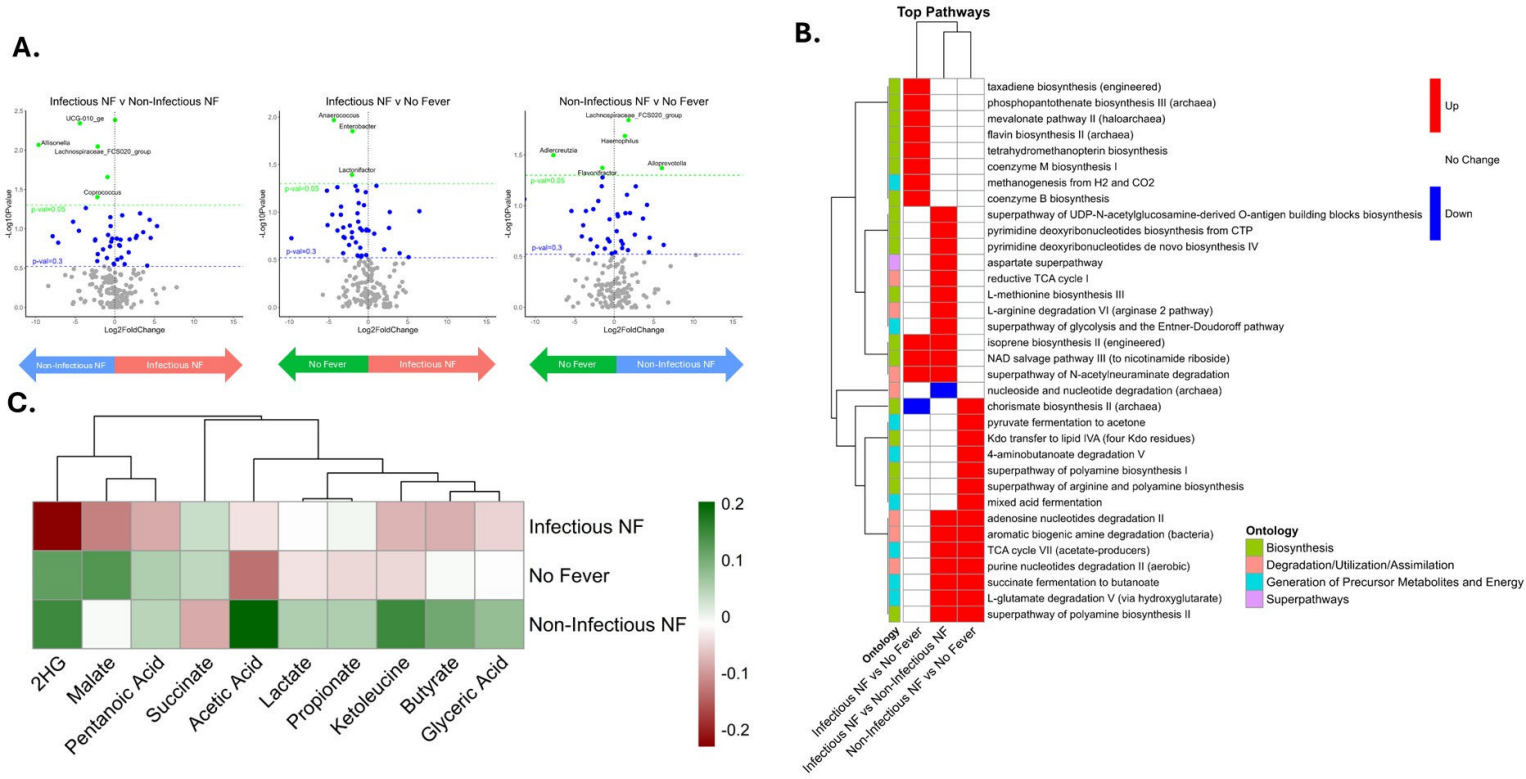
Differential abundance analysis of composition and function of the baseline gut microbiome. (**A**) Volcano plots of estimated log2 fold difference in abundance between infectious NF and non-infectious NF (left), infectious NF and no fever (middle), and non-infectious NF and no fever (right). Green and blue horizontal lines indicate 0.05 and 0.3 *P* value thresholds. Volcano plots are shown with a horizontal threshold at *P* = 0.3 to visually highlight which features are retained for multivariate modeling. (**B**) Heatmap depicts log2 fold changes (L2FC) of pathways identified using MetaCyc, analyzed with LinDA. Rows represent individual pathways, clustered by similarity, and columns represent pairwise comparisons. Color indicates direction and significance of change: red denotes a significant increase in the first group listed compared to the second group (*P* ≤ 0.1, L2FC > 0.3); blue indicates a significant decrease in the first group listed compared to the second group (*P* ≤ 0.1, L2FC > −0.3), and white shows no significant change (*P* > 0.1). Pathways are annotated with their associated ontology. (**C**) Heatmap of Spearman correlation coefficients between metabolites and outcomes. Colors represent the correlation value, with green being more positive and red being more negative. Abbreviations: 2HG, 2-hydroxyglutarate; LinDA, linear regression framework for differential abundance analysis; NF, neutropenic fever.

We next conducted pairwise comparisons of genera between patient groups to identify taxonomic shifts associated with infection status and to support downstream feature selection for predictive modeling. These results were visualized using volcano plots to highlight taxa with both statistical significance and biologically meaningful effect size (Fig. 2A; Tables S8 to S10).

At baseline, six genera were differentially abundant between the infectious and non-infectious NF groups (Fig. 2A; Table S8). Notably, several short-chain fatty acid (SCFA)-producing taxa, including *Lachnospiraceae_FCS020_group*, *Lachnospira*, and *Coprococcus*, were significantly more abundant in the non-infectious NF group. Comparing infectious NF to no fever, three genera*—Anaerococcus*, *Enterobacter*, and *Lactonifactor*—were significantly more abundant in the no-fever group (Table S9). However, *Enterobacter* reads were detected in only 5 out of 23 no-fever patients and absent in 14 out of 33 infectious NF patients, suggesting that this signal may be driven by a small number of individuals. Between non-infectious NF to no fever, five genera differed significantly in abundance, with *Lachnospiraceae_FCS020_group*, *Haemophilus*, and *Alloprevotella* having a higher relative abundance in non-infectious NF, and *Adlercreutzia* and *Flavonifractor* being more abundant in individuals with no fever. (Table S10).

PICRUSt2 analysis was performed to predict bacterial functions, revealing predicted differences in microbial pathways between fever groups at baseline. Compared to the no-fever group, the infectious NF group showed a higher predicted abundance of pathways involved in “biosynthesis” (Fig. 2B). When comparing infectious NF to non-infectious NF, the infectious group also had increased predicted pathways related to both biosynthesis and “generation of precursor metabolites and energy.” In the comparison between non-infectious and no fever, pathways under the “degradation/utilization, and assimilation” ontology were more abundant in the non-infectious NF group. Specific predicted pathways such as “succinate fermentation to butanoate” and “TCA cycle VII (acetate producers)” were enriched in infectious NF compared to non-infectious NF and also enriched in non-infectious NF compared to no-fever (Fig. 2B).

To better characterize baseline metabolite profiles across outcome groups, we performed targeted fecal metabolomics using IC-MS (Table S11; Fig. S2) and assessed associations between metabolite levels and fever outcomes using Spearman correlation. Notably, 2HG at baseline was negatively correlated with infectious NF, suggesting that lower levels of this metabolite are associated with infectious NF. In contrast, acetic acid was positively correlated with non-infectious NF, indicating higher levels may be linked to non-infectious inflammatory responses (Fig. 2C). These results support the idea that distinct metabolic signatures may underlie the differences between infectious and non-infectious NF outcomes.

To explore potential interactions among microbial taxa, metabolites, and clinical features at baseline, we performed integrated network analysis separately for patients with infectious NF (Fig. S3A) and non-infectious NF (Fig, S3B). The infectious NF baseline network comprised 56 nodes and 99 edges, with a mean degree of 1.768, density of 0.064, average clustering coefficient of 0.447, and a modularity of 0.548. In comparison, the non-infectious NF network included 171 nodes and 630 edges, mean degree 3.684, density 0.043, average clustering coefficient 0.519, and modularity 0.449. Thus, the infectious NF network exhibited a very similar degree of connectivity and modularity compared to the non-infectious NF network.

### Variable selection and machine-learning model performance for baseline prediction of infectious vs non-infectious NF

We applied a supervised machine learning approach to predict infectious vs non-infectious NF using baseline microbial, metabolite, and clinical features, with models trained on variables selected through four distinct selection methods (Table S12). Model A included all raw variables without transformation; model B applied CLR transformation to microbial variables; model C applied univariate filtering based on statistical tests (*χ*^2^, Fisher’s Exact, and Mann–Whitney) to retain variables with *P* < 0.3; and model D applied CLR transformation to microbial variables retained in model C. These methods included CLR transformation and variable thresholding to improve interpretability, reduce overfitting, and eliminate irrelevant variables.

**Fig 3:**
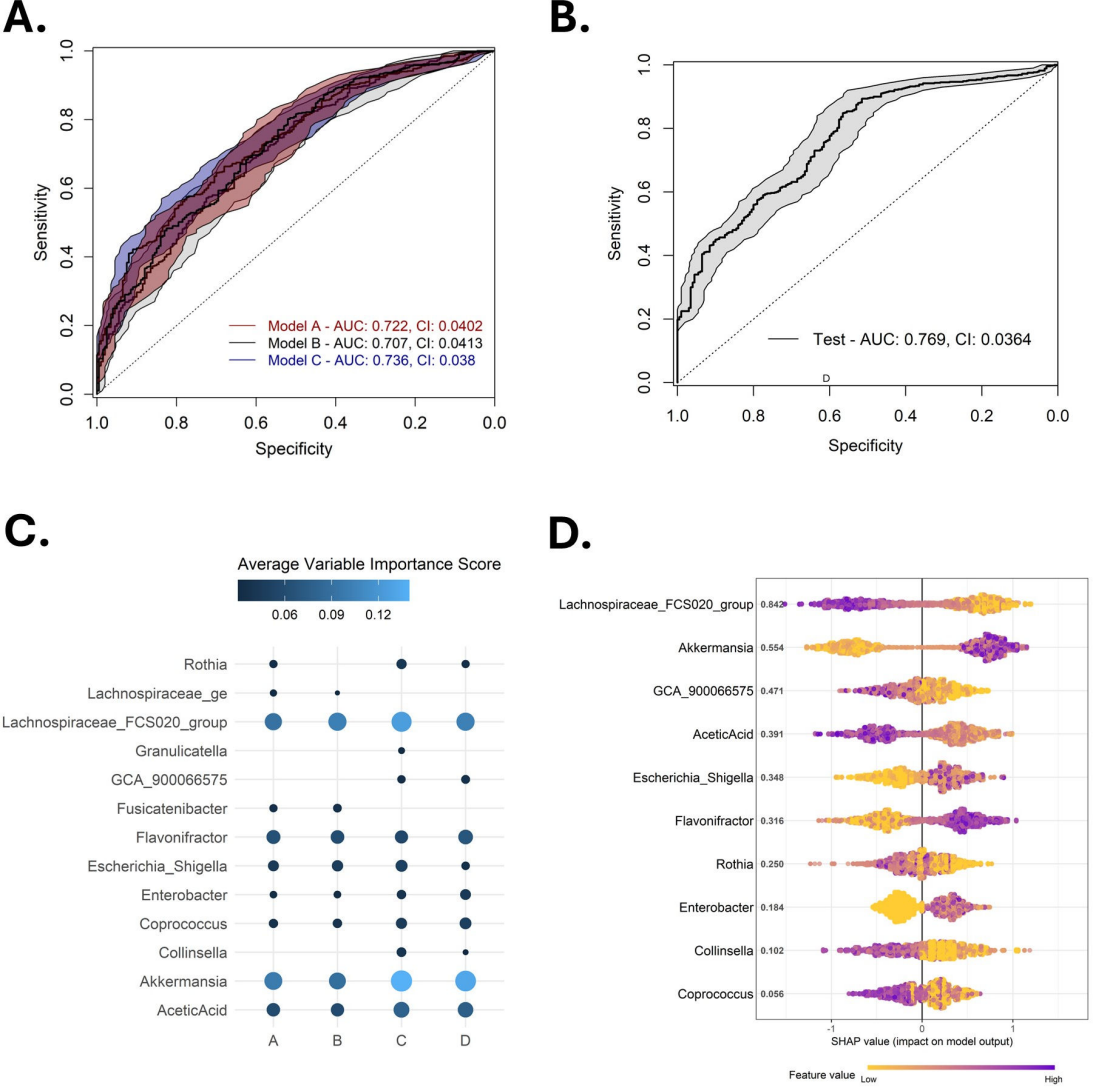
Predictive performance and biomarker discovery of the baseline model distinguishing infectious from non-infectious NF. (**A**) Graphic representation of ROC curves for three initial variable selection methods (models A, B, and C). (**B**) ROC curve for the final selected model (Model D). Solid lines indicate the mean ROC curve, while shaded regions represent the 95% confidence intervals. AUC and confidence intervals are printed on the plot. (**C**) Bubble plot of variable importance scores for the four variable selection models. Size and colors indicate the importance score, where lighter larger bubbles indicate a higher variable importance score. (**D**) Features and their importance computed through SHAP analysis. Variables are ordered according to importance from top to bottom with SHAP values printed on the plot. The *X*-axis depicts the SHAP value indicating the change in log odds, where a larger SHAP value indicates a positive influence on the outcome of infectious NF. Each point represents a single sample. Values on the *Y*-axis represent the mean absolute SHAP value for each feature across all samples. The gradient color indicates the original value for that variable. Abbreviations: AUC, area under the curve; CI, confidence interval; ROC, receiver operator curve; SHAP, Shapley additive explanations.

When comparing these variable selection methods, we observed that models using variable thresholding (models C and D) yielded the highest AUROC values (Fig. 3A and B; Fig. S4). Reducing the number of variables from model A (all variables; aggregate AUROC = 0.722, 95% confidence interval [CI] 0.0402, SD = 0.161, variance = 0.0259, average precision = 0.625) to model C (*P* ≤ 0.3; aggregate AUROC = 0.736, CI 0.038, SD = 0.162, variance = 0.0263, average precision = 0.625) showed minor improvement in discrimination, suggesting that the elimination of noise and irrelevant features contributed to model performance. A similar trend was observed between model B (aggregate AUROC = 0.707, CI 0.0413, SD = 0.174, variance = 0.0303, average precision = 0.629) and model D (AUROC = 0.769, CI 0.0364, SD = 0.162, variance = 0.0263, average precision = 0.612), which underwent the same elimination process.

Interestingly, CLR transformation did not consistently improve model performance but resulted in a reduced performance in model B compared to model A (Fig. 3A). The best performance was achieved by model D (AUROC = 0.769), which combined CLR-transformed genera abundances with variable selection based on univariate filtering at *P* < 0.3. Biomarkers were selected from each model based on a mean variable importance score of ≥0.3. Notably, the selected biomarkers were largely consistent across models, and CLR transformation had minimal impact on the final set of features (Fig. 3C; Table S13).

Finally, SHAP analysis was performed to estimate the marginal contribution of each variable to the final model’s prediction (Fig. 3D). This analysis revealed that higher CLR-transformed abundances of *Akkermansia*, *Enterobacter*, *Escherichia*–*Shigella*, and *Flavonifractor* contributed to shifting the model’s prediction toward infectious NF. In contrast, features such as acetic acid and SCFA-producing bacteria, including *Collinsella*, *Coprococcus*, and GCA-900066575 (Lachnospiraceae family), contributed toward the model’s prediction of non-infectious NF. Several variables exhibited non-linear effects, highlighting the complexity of abundance patterns in shaping the classification decision.

### Distinct microbial and functional profiles differentiate infectious from non-infectious NF at the onset of fever

**Fig 4:**
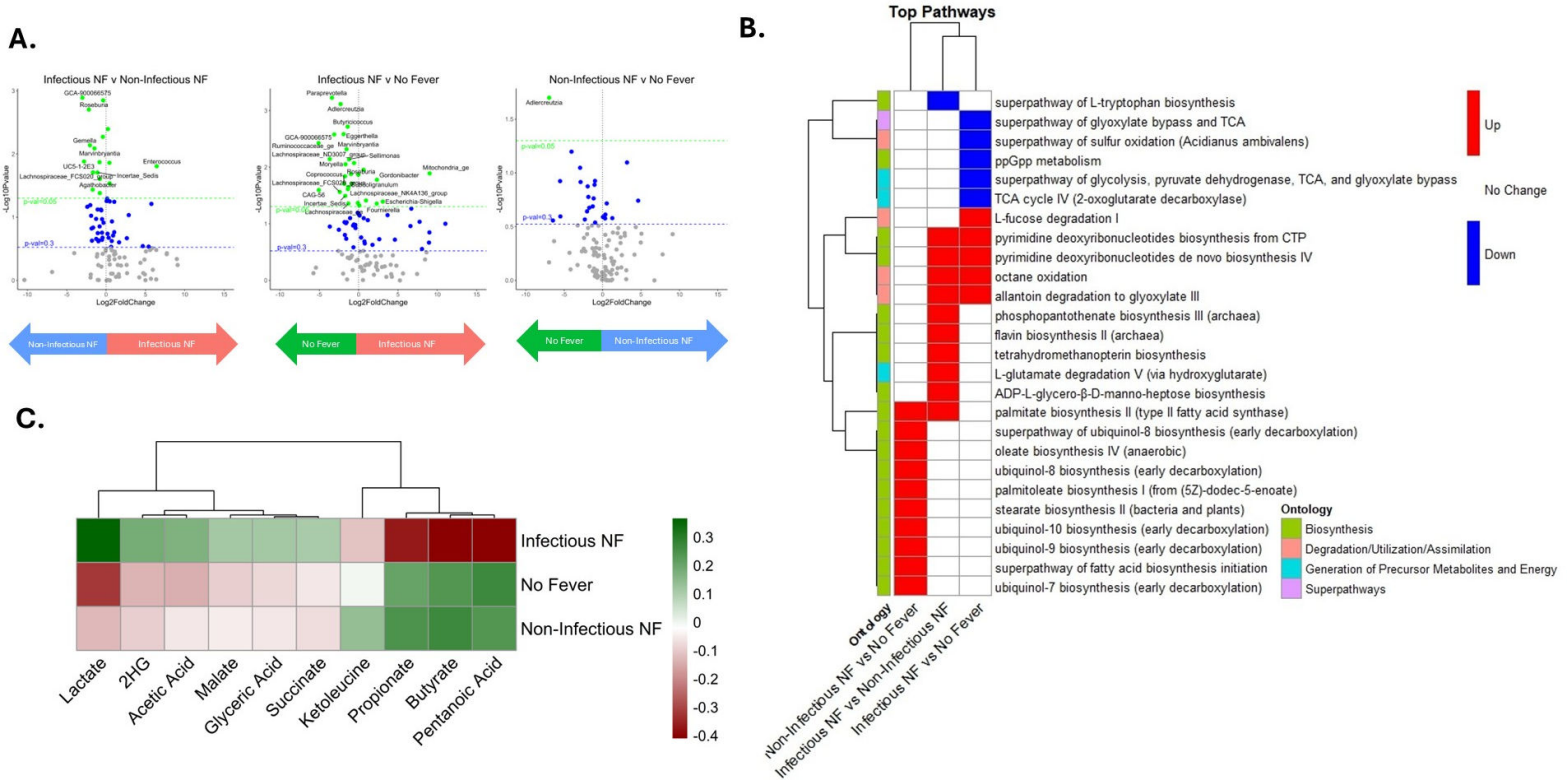
Differential abundance analysis of composition and function of onset gut microbiome. (**A**) Volcano plots of estimated log2 fold difference in abundance between infectious NF and non-infectious NF (left), infectious NF and no fever (middle), and non-infectious NF and no fever (right). Green and blue horizontal lines indicate 0.05 and 0.3 *P* value thresholds. Volcano plots are shown with a horizontal threshold at *P* = 0.3 to visually highlight which features were retained for multivariate modeling. (**B**) Heatmap depicts log2 fold changes of pathways identified using MetaCyc, analyzed with LinDA. Rows represent individual pathways, clustered by similarity; columns represent pairwise comparisons. Color indicates direction and significance of change: red denotes a significant increase in the first group listed compared to the second group (*P* ≤ 0.1, L2FC > 0.3); blue indicates a significant decrease in the first group listed compared to the second group (*P* ≤ 0.1, L2FC > −0.3); and white shows no significant change (*P* > 0.1). Pathways are annotated with their associated ontology. (**C**) Heatmap of Spearman correlation coefficients between metabolites and outcomes. Colors represent correlation, with green being more positive and red being more negative. Abbreviations: 2HG, 2-hydroxyglutarate; LinDA, linear regression framework for differential abundance analysis; NF, neutropenic fever.

To complement our baseline analyses focused on prediction, we next examined gut microbiome composition at the onset of NF to gain insight into the biological differences between fever events. A total of 17 genera exhibited significantly different relative abundances between infectious and non-infectious NFs, with 13 more abundant in non-infectious NF and 4 enriched in infectious NF (Fig. 4A; Table S14). Notably, several taxa enriched in non-infectious NF—*Lachnospiraceae NK4A136* group, *Roseburia*, and *Coprococcus*—are known for their roles in SCFA production. In contrast, genera more abundant in infectious NF included *DTU089*, *Ruminococcaceae_unclass*, *Enterococcus*, and *Faecalibacterium*.

When comparing infectious NF to no fever, 29 genera were significantly different (Fig. 4A; Table S15). Six genera, including DTU089, *Gordonibacter*, and *Escherichia*–*Shigella*, were more abundant in infectious NF, whereas 23 genera, including *Paraprevotella*, Lachnospiraceae_ND3007_group, and Ruminococcaceae_ge, were more enriched in the no-fever group. Finally, a single genus distinguished non-infectious NF from no fever, *Adlercreutzia* was significantly more abundant in the no-fever group (Fig. 4A; Table S16).

Analysis of inferred metabolic pathway differences between infectious and non-infectious NFs using PICRUSt2 revealed distinct predicted patterns of functional shifts across metabolic ontologies (Fig. 4B). At fever onset, pathways within the biosynthesis and degradation/utilization/assimilation ontologies were significantly more abundant in infectious NF compared to non-infectious NF. In contrast, comparisons between non-infectious NF and no fever only showed significant differences in biosynthesis pathways. Specifically, non-infectious NF exhibited increases in multiple predicted biosynthetic pathways, including “oleate biosynthesis IV (anaerobic),” “palmitate biosynthesis II (type II fatty acid synthase),” and “ubiquinol-8 biosynthesis (early decarboxylation).” Interestingly, infectious NF was associated with reduced abundance of several central metabolic pathways compared to both non-infectious NF and no fever, including “superpathway of L-tryptophan biosynthesis,” “ppGpp metabolism,” and “superpathway of glycolysis, pyruvate dehydrogenase, TCA, and glyoxylate bypass.” This downregulation of predicted core energy-generating pathways may reflect a shift away from aerobic energy metabolism.

Stool metabolite analysis at fever onset revealed distinct metabolic signatures associated with each outcome group (Table S17; Fig. S5). Six metabolites, including lactate, 2HG, acetic acid, malate, glyceric acid, and succinate, were positively correlated with infectious NF but negatively correlated with both non-infectious NF and no fever, with lactate showing the strongest association (Fig. 4C). Conversely, four metabolites, butyrate, propionate, pentanoic acid, and ketoleucine, were positively correlated with no fever and non-infectious fever, but negatively correlated with infectious NF.

### Microbial and metabolite biomarkers differentiate infectious from non-infectious NF at fever onset

**Fig 5:**
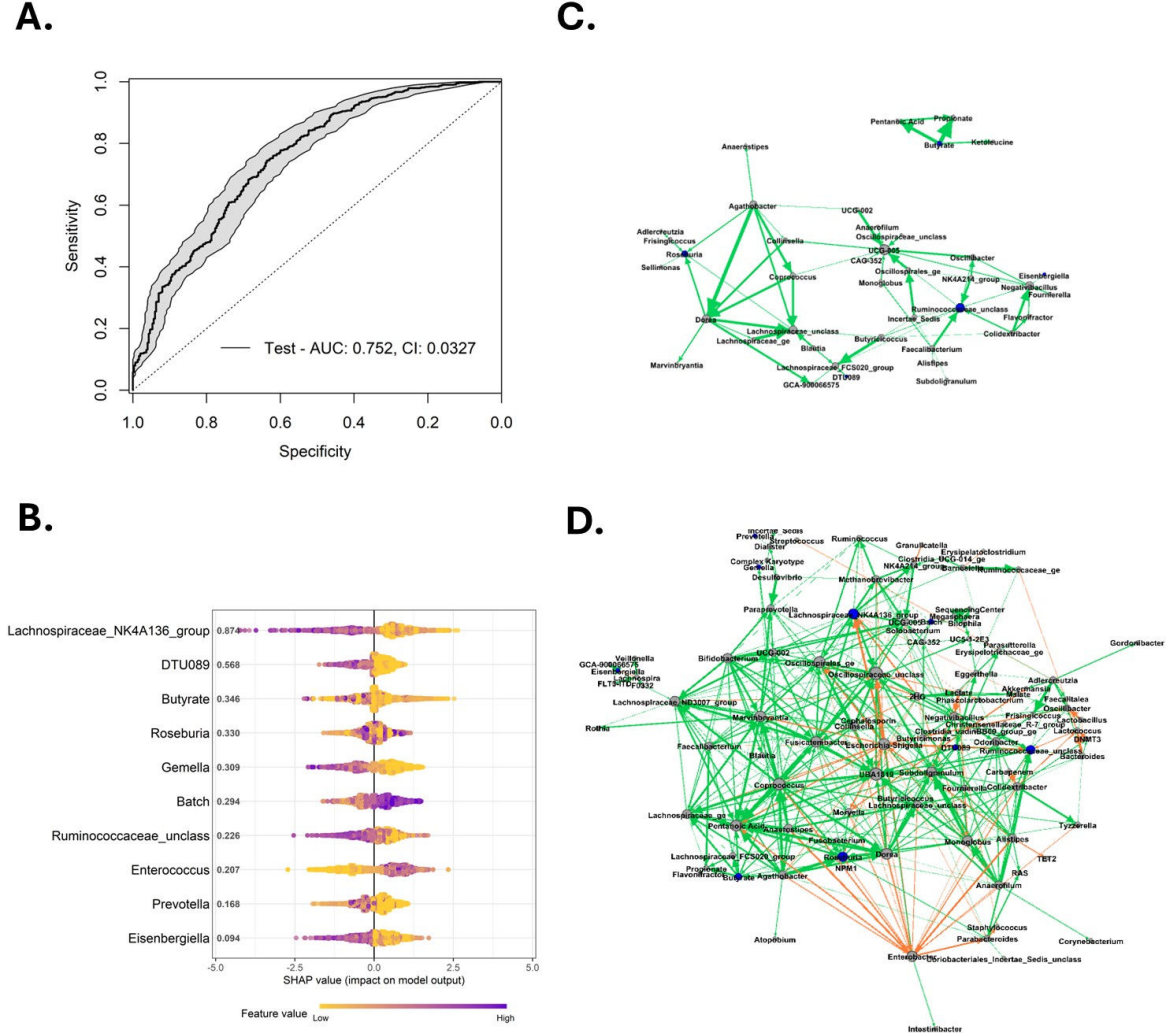
Microbial and metabolite biomarkers differentiate between infectious and non-infectious NFs at onset. (**A**) ROC of the model differentiating infectious and non-infectious NFs at fever onset. Solid lines indicate the mean ROC curve, while shaded regions represent the 95% CI. AUC and confidence intervals are printed on the plot. (**B**) Features and their importance computed through SHAP analysis. Variables are ordered according to importance from top to bottom with SHAP values printed on the plot. The *X*-axis depicts the SHAP value indicating the change in log odds, where a larger SHAP value indicates a positive influence on the outcome of infectious NF. Each point represents a single sample. Values on the *Y*-axis represent the mean absolute SHAP value for each feature across all samples. The gradient color indicates the original value for that variable. Integrated network analysis of microbes, metabolites, and clinical variables for infectious NF patients (**C**) and non-infectious NF patients (**D**). Each variable is represented by different nodes, the size of which represents their relative abundance. Blue nodes represent biomarkers identified in the onset model (**A**). Edge thickness represents the correlation coefficient. Green lines represent a positive correlation, and red lines represent a negative correlation. Abbreviations: AUC, area under the curve; CI, confidence interval; ROC, receiver operator curve; SHAP, Shapley additive explanation.

To identify biomarkers that differentiate non-infectious and infectious NF at fever onset, we developed an ML model using the same preprocessing approach that yielded the best performance in our baseline analysis: univariate variable selection followed by CLR transformation of microbial variables. This model achieved an AUROC of 0.752 (95% CI ±0.0327, SD = 0.166, variance = 0.0275, average precision = 0.607) (Fig. 5A; Fig. S6). SHAP analysis was used to interpret the contribution of individual features to the model’s predictions (Fig. 5B, Fig. S6). *Enterococcus* was the only microbial variable whose high feature values contributed positively toward the outcome of infectious NF, indicating its presence increased the model’s likelihood of classifying a sample as infectious NF. In contrast, *Lachnospiraceae_NK4A136_*group, *Gemella*, DTU089, *Ruminococcaceae_unclass*, and *Eisenbergiella* contributed to predictions favoring non-infectious NF.

Because onset samples were collected within a window spanning several days before and after NF, we evaluated whether antibiotic escalation at fever onset confounded our findings. Stratifying onset samples by pre-antibiotic vs post-antibiotic collection showed no significant differences in weighted or unweighted UniFrac β-diversity, and the relative abundances of onset model biomarkers were similar between groups, with the exception of butyrate (Fig. S7). These results indicate that the key microbial and metabolite features identified at fever onset are unlikely to be driven solely by the timing of sample collection relative to antibiotic modification.

To further investigate the microbial and metabolic interactions shaping these phenotypes, we performed network analysis based on correlations between microbial, metabolite, and clinical variables. In infectious NF (Fig. 5C), *Ruminococcus_unclass* and *UCG-005* emerged as central nodes. Strong positive correlations were observed among SCFA-producing bacteria, while metabolites like butyrate and propionate formed a separate network. The infectious NF network was sparse and fragmented, consisting of just 42 nodes and 66 edges (mean degree 1.571, density 0.077) with an average clustering coefficient of 0.421 and higher modularity (0.514), reflecting strong compartmentalization into modules and a lack of overall connectivity.

The network in non-infectious NF displayed a much denser and more interconnected structure, with 102 nodes and 439 edges (mean degree 4.304, density 0.085, clustering coefficient 0.448, modularity 0.485), suggesting a more functionally diverse microbiome and metabolic network (Fig. 5D). SCFA-producing taxa (*Ruminococcus*, *Coprococcus*, and *Lachnospiraceae_NK4A136*_*group*) and metabolites like butyrate, pentanoic acid, and lactate were central nodes, indicating preserved microbial function during non-infectious NF events. Positive correlations among SCFAs and their producing taxa suggest functional stability within the microbiota despite inflammatory perturbations in non-infectious NF.

## DISCUSSION

Despite extensive research, distinguishing infectious from non-infectious NF in hematologic cancer patients remains a persistent challenge, often leading to unwarranted use of broad-spectrum antibiotics. The empirical treatment upon NF onset, often without de-escalation after microbial culture results, contributes to rising antimicrobial resistance and profound gut microbiome disruption, highlighting the urgent need for better diagnostic tools and improved antimicrobial stewardship rooted in a clearer understanding of NF pathophysiology. In this study, we used a machine learning and network-based approach to integrate gut microbiome, fecal metabolomics, and clinical features, revealing distinct microbial and metabolic profiles that differentiate non-infectious from infectious NF in AML patients.

One of the most striking findings was the clear ecological and functional divergence between non-infectious and infectious NF. Across both baseline and fever onset, the non-infectious NF microbiome maintained a highly interconnected network structure characterized by greater density, clustering, and mean degree, which may signify robust functional redundancy and compositional stability within the microbial ecosystem. In contrast, the infectious NF network exhibited marked fragmentation at fever onset, revealing far fewer microbial connections, a mean degree well below 1, zero clustering coefficient, and significantly higher modularity. These patterns suggest that non-infectious NF is associated with a resilient and metabolically cooperative microbial ecosystem, while infectious NF reflects ecological instability and disrupted microbial interactions. Interestingly, previous research has shown that microbiome stability during transplantation correlates with shorter fever durations in pediatric allogenic HSCT patients (32).

*Allisonella*, *Lachnospira*, and *Coprococcus* were significantly enriched in non-infectious NF at baseline, while genera within the Lachnospiraceae family, including *Coprococcus*, *Roseburia*, and *Dorea*, were significantly enriched in non-infectious NF at onset, as revealed by the two-group comparisons. These microbes are known to generate SCFAs, including butyrate and acetate, which play critical roles in maintaining gut barrier integrity and regulating inflammation. This finding aligns with previous research in pediatric acute lymphoblastic leukemia (ALL) patients, which linked the loss of SCFA-producing Lachnospiraceae members to prolonged neutropenia (33). Supporting this further, a recent study of pediatric patients with antibiotic-associated neutropenia reported a significant decrease in the abundance of Lachnospiraceae and corresponding depletion of gut-derived microbial metabolites (34).

Our baseline predictive model identified *Akkermansia* as a strong predictor of infectious NF. While traditionally associated with gut health, emerging data indicate that its effects are context dependent. In metabolically healthy hosts, moderate *Akkermansia* abundance has been linked to improved metabolic and barrier function (35, 36). However, under dysbiotic conditions (i.e., chemotherapy-induced epithelial injury and broad-spectrum antibiotic exposure), excessive expansion of mucin-degrading bacteria may become maladaptive, leading to degradation of the mucosal barrier, potentially promoting pathogen translocation and febrile responses (37, 38). These findings align with several studies that have reported increased *Akkermansia* abundance at fever onset in HSCT patients, as well as with studies showing that its higher abundance is predictive of a higher risk of NF (17, 32) (12). Importantly, recent work by Schwabkey et al. demonstrated that *Akkermansia muciniphila* expansion following cytotoxic therapy in murine models resulted in mucus layer thinning, increased flagellin translocation, and elevated colonic inflammation, culminating in impaired thermoregulation and fever (17).

On the other hand, *Enterococcus* abundance was a major contributor to the fever onset model performance, with higher levels strongly influencing the model’s classification toward infectious NF. *Enterococcus* has recently emerged as potentially pro-inflammatory in the context of gut dysbiosis and has been associated with bacteremia and systemic inflammation (39). Several publications have implicated *Enterococcus* overgrowth in NF settings, reporting significant increases compared to pre-treatment levels in patients with AML and pediatric ALL (13, 33). Additionally, a recent study demonstrated that higher pre-chemotherapy *Enterococcus* abundance predicted prolonged chemotherapy-induced neutropenia in leukemia patients (40).

Metabolomic profiling further reinforced the microbial distinctions between NF types. The metabolites profiled in this study were selected to capture key microbiota-derived and host-associated pathways relevant to epithelial integrity and inflammation. SCFAs, such as butyrate, propionate, and pentanoic acid, serve as energy substrates for colonocytes and upregulate tight-junction proteins, thereby strengthening the intestinal barrier and dampening pro-inflammatory signaling (39, 40). SCFAs also inhibit the NF-kB pathway, a key driver of inflammation that is activated during chemotherapy-induced gut barrier injury (33, 34). Additionally, organic acids involved in central carbon metabolism (lactate, malate, succinate, glyceric acid, and ketoleucine) and 2-HG have established links to epithelial barrier integrity, inflammation, and host energy metabolism (41–43). Infectious NF was associated with elevated levels of lactate, acetic acid, and 2HG, metabolites commonly linked to anaerobic metabolism and oxidative stress. In contrast, butyrate, propionate, and pentanoic acid were negatively associated with infectious NF. The inverse relationship between propionate and infectious NF is particularly notable, given recent findings that propionate supplementation in murine models of chemotherapy-induced mucosal injury mitigated fever by preserving the mucus barrier and reducing inflammatory cytokine production (17). These metabolic patterns reflect not only shifts in microbial composition but also fundamental differences in host–microbe interactions during fever, with potential implications for future interventions targeting metabolic pathways to restore gut homeostasis.

However, this study has several limitations. As a single-center study, our findings may not be generalizable to other institutions or geographic regions; thus, external validation in independent cohorts is essential to confirm generalizability. A selection bias inherent to human population studies is also acknowledged as stool samples were only collected from consenting patients. Our use of 16S rRNA sequencing restricts taxonomic resolution to primarily the genus level; therefore, a more comprehensive understanding of microbial and metabolic dynamics would require deeper characterization, including full metabolomic profiling and more granular sequencing approaches. There also remains a risk of misclassification among culture-negative patients due to limitations in clinical diagnostics and decision-making at the hospital. We also recognize the absence of important clinical metadata (mucositis grade, CVC status, gastrointestinal symptoms, diet, etc.) that can influence microbiome and metabolome profiles and serve as confounding variables. These variables were not collected in our cohort and thus could not be considered in our analysis. Moreover, given the high number of statistical comparisons performed, although many taxa reached nominal significance (*P* < 0.05), none remained significant after BH correction for multiple testing. This outcome is common in high-dimensional microbiome studies, where controlling for false discovery rate can be highly conservative, particularly when true effects are modest or sample size is limited. As such, our findings should be viewed as exploratory and hypothesis generating, with larger cohorts needed to clarify the biological relevance of the observed associations. Future work should include longitudinal sampling, functional validation of key taxa, and interventional studies to test microbiome-targeted therapies aimed at preventing or mitigating NF.

Despite these limitations and the reported AUROC values for the baseline and fever onset models having modest predictive power in differentiating infectious from non-infectious NF, both models do indeed demonstrate clinically relevant classification capabilities. This suggests the potential utility of microbiome profiling as part of routine assessments for febrile risk and infection susceptibility early on. Although several machine learning models have been developed to assess infection risk in NF patients, they have largely relied on clinical variables without incorporating microbiome or metabolite data (41–44). Thus, our integrative model expands current methodologies by incorporating microbial and metabolic profiles, opening new avenues for risk stratification and personalized intervention. Together, our findings highlight distinct microbial ecosystems and metabolic adaptations underlying infectious and non-infectious NFs, providing a framework for the development of diagnostic tools rooted in gut ecology. The identification of both bacterial and metabolic signatures offers potential targets for clinical risk stratification and therapeutic modulation.

## Data Availability

16S rRNA sequences can be found in the NCBI Sequence Read Archive (http://www.ncbi.nlm.nih.gov/sra) under the BioProject IDs PRJNA352060 and PRJNA526551 (PA13-0339) and PRJNA1124986 (PA15-0780). The code is available in the GitHub repository at https://github.com/SRFranklin7/NF-infection-model/ and archived with the DOI https://doi.org/10.5281/zenodo.15634065 under the MIT license.

## SUPPLEMENTAL MATERIAL

Supplemental material msystems.01343-25-s0001.pdf Fig. S1-S7 and Tables S1-S17.

ASM does not own the copyrights to Supplemental Material that may be linked to, or accessed through, an article. The authors have granted ASM a non-exclusive, world-wide license to publish the Supplemental Material files. Please contact the corresponding author directly for reuse.
