# Supplementary material for: Short-chain fatty acid-producing microbes differentiate non-infectious and infectious neutropenic fever in leukemia
Source: mSystems. 2026 Mar 31;11(4):e01343-25. doi: 10.1128/msystems.01343-25 (PMC13098215; doi:10.1128/msystems.01343-25)
Supplement: Supplemental material — Fig. S1-S7 and Tables S1-S17. [file msystems.01343-25-s0001.pdf]

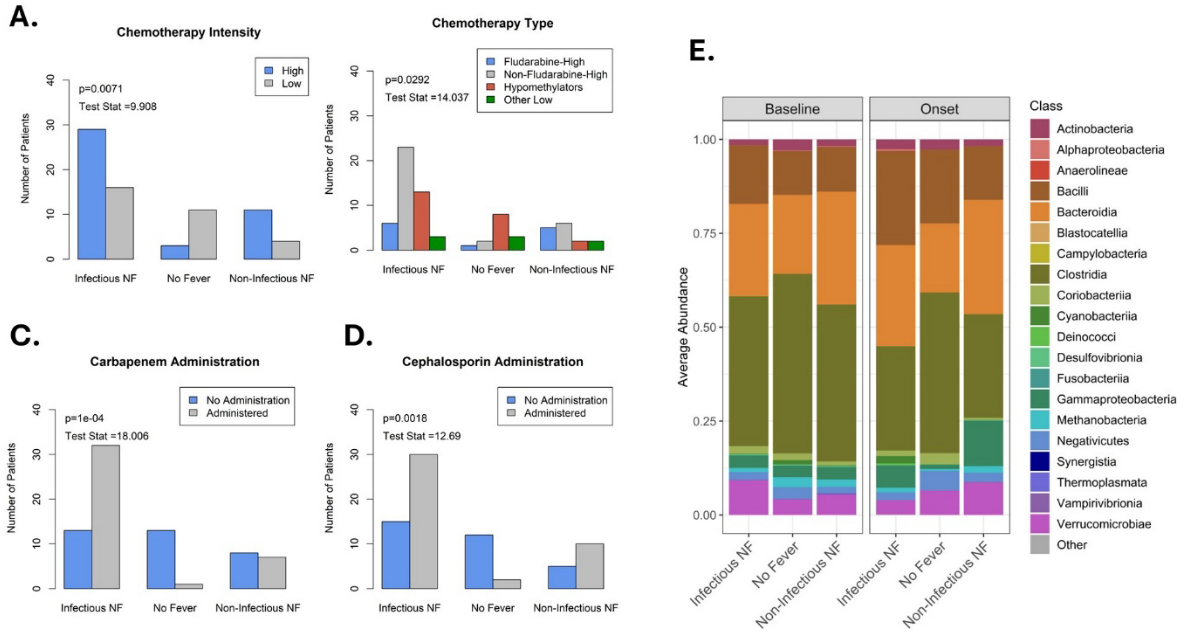

Supplemental Figure 1. Distribution of clinical variables and class abundances within patients. Bar plots displaying the distribution of chemotherapy intensity (A), chemotherapy type (B), carbapenem administration (C), and cephalosporin administration (D) among patients included in the onset analysis grouped by outcome. Bar height indicates patient counts and annotated with Chi-squared test-statistic and p-value. (E) Barplot of the average relative abundance of the microbial classes present within each outcome at baseline (left) and onset of neutropenic fever (right).

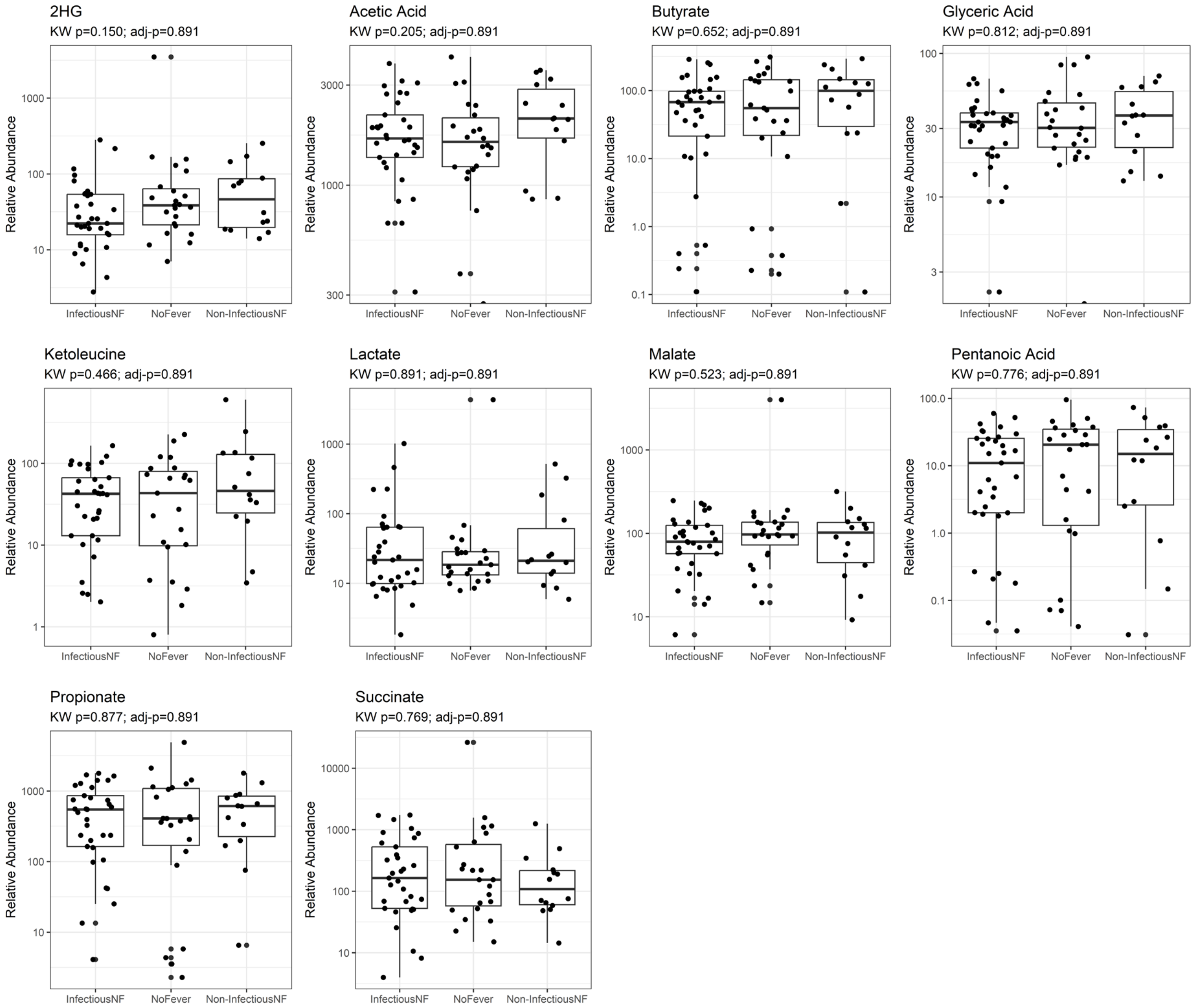

Supplemental Figure 2: Boxplots of $\log_{10}$-transformed metabolite abundances across treatment groups at baseline. Abundances of fecal metabolites are shown on the y-axis, each point represents the metabolite abundance of a single patient. Unadjusted and BH corrected p-values from a Kruskal-Wallis test is shown above each plot, comparing metabolite abundance across treatment groups.

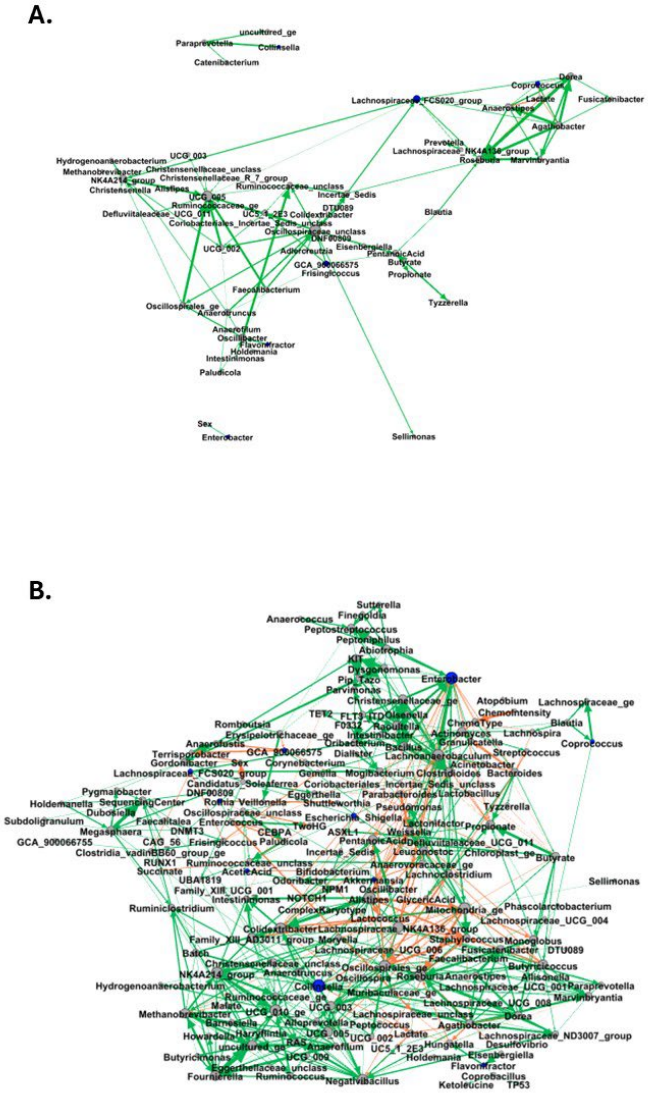

Supplemental Figure 3: Microbial and metabolite network of infectious and non-infectious NF at baseline. Integrated network analysis of microbes, metabolites, and clinical variables for infectious NF (A) and non-infectious NF (B) patients. Each variable is represented by different nodes, the size of which represents their relative abundance. Blue nodes represent biomarkers identified in the baseline model. Edge thickness represents the correlation coefficient. Green lines represent positive correlation, and red lines represent negative correlation.

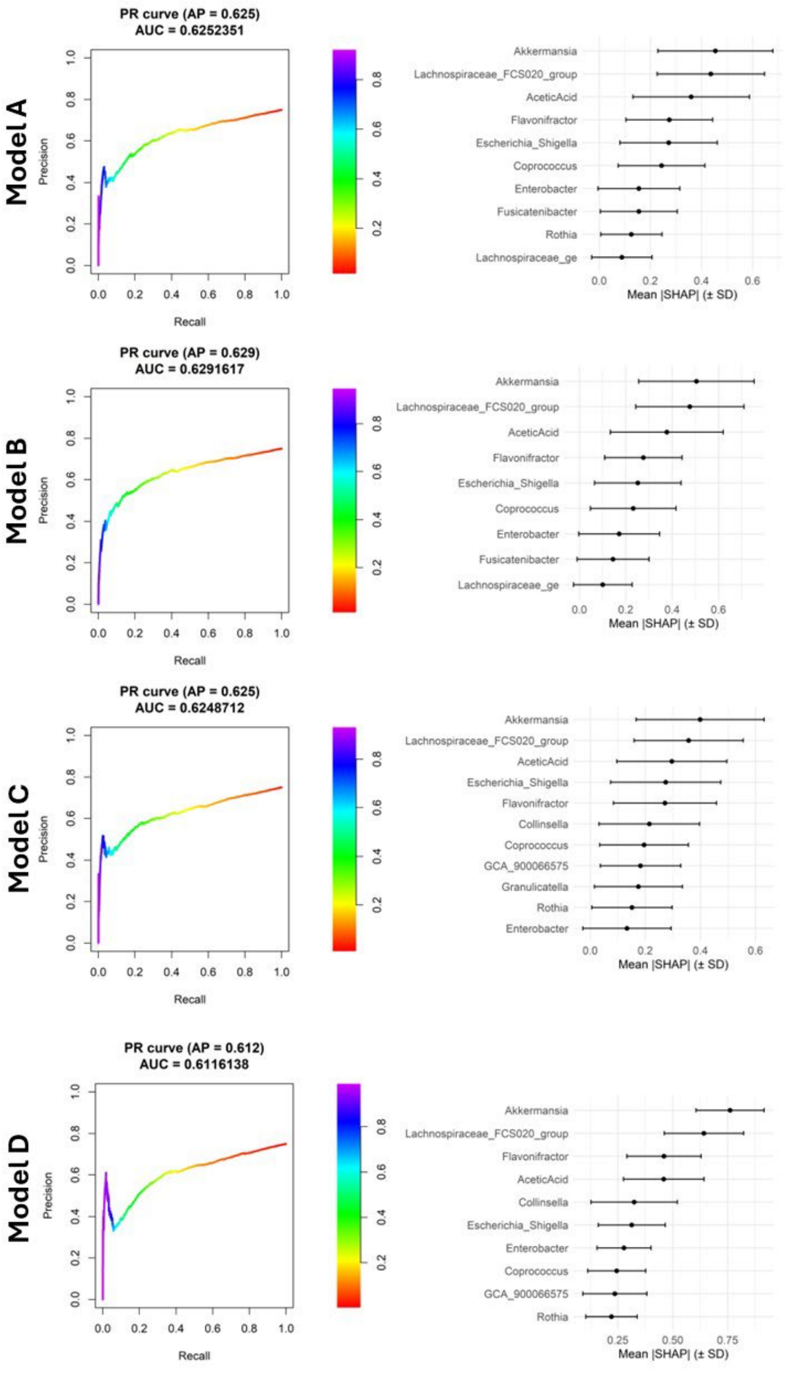

Supplemental Figure 4: Precision–recall curves and baseline SHAP feature importance across XGBoost models. For each model (Model A–D), the left panel shows the precision–recall (PR) curve with area under the PR curve (average precision, AP) and area under the ROC curve (AUC) summarizing discrimination performance for predicting infectious versus non-infectious NF. The right panel displays the mean absolute SHAP value (± SD) across 100 bootstrap iterations for the top microbiome and metabolite features in each model, representing their average contribution to model predictions and the stability of their importance estimates.

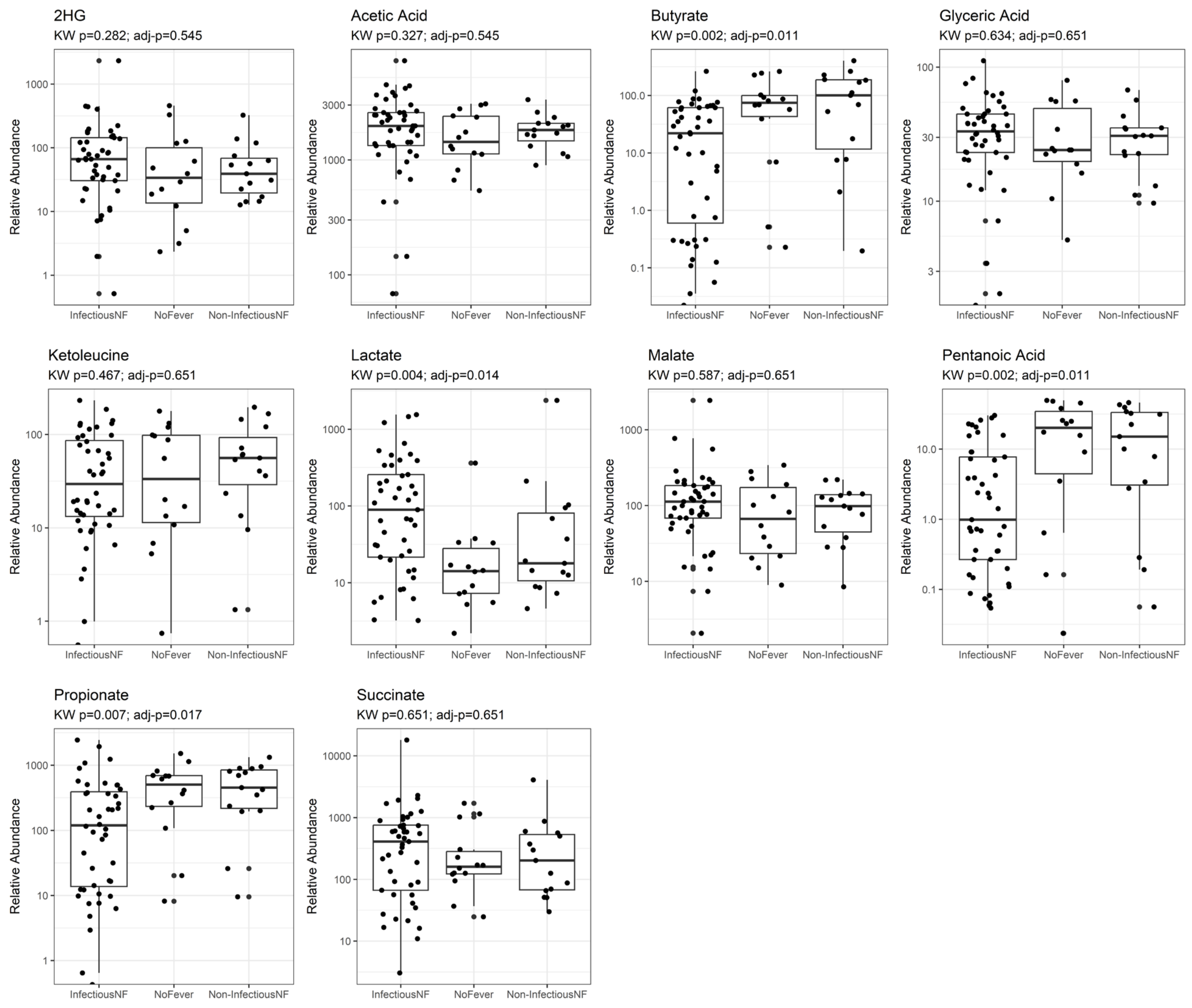

Supplemental Figure 5: Boxplots of log_{10}-transformed metabolite abundances across treatment groups at onset of fever. Abundances of fecal metabolites are shown on the y-axis, each point represents the metabolite abundance of a single patient. Unadjusted and BH corrected p-values from a Kruskal-Wallis test is shown above each plot, comparing metabolite abundance across treatment groups.

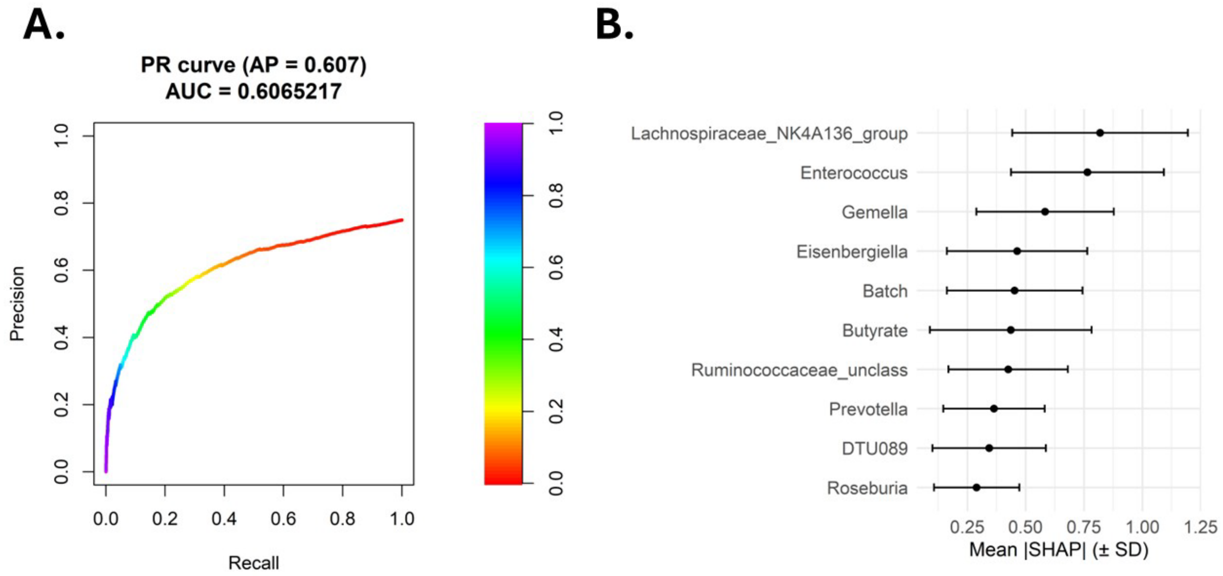

Supplemental Figure 6: Precision–recall curve and onset SHAP feature importance. (A) Precision-recall (PR)curve with area under the PR curve (average precision, AP), and area under the ROC curve (AUC) summarizing the discrimination performance for predicting infectious versus non-infectious NF. (B) Mean absolute SHAP value (± SD) across 100 bootstrap iterations for the top microbiome and metabolite features in the onset model, representing their average contribution to model predictions and the stability of their importance estimates.

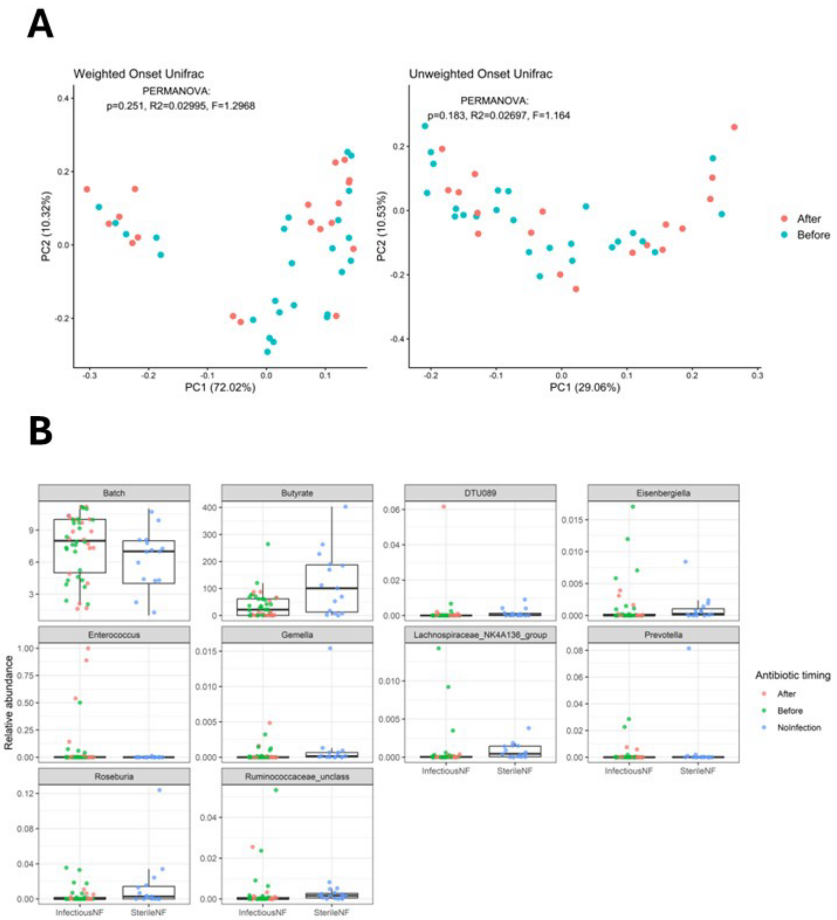

C

Variable	P-value (unadjusted)
Batch	0.755
Butyrate	0.0514
DTU089	0.976
Eisenbergiella	0.319
Enterococcus	0.971
Gemella	0.402
Lachnospiraceae_NK4A136_group	0.208
Prevotella	0.364
Roseburia	0.167
Ruminococcaceae_unclass	0.452

Supplemental Figure 7: Sensitivity analysis of onset samples in relation to antibiotic timing. (A) Principal coordinate analysis (PCoA) plots of weighted (left) and unweighted (right) UniFrac distances for onset stool samples, colored by timing of collection relative to fever-associated antibiotic escalation (“Before” in blue; “After” in red). Each point represents a patient with infectious NF, and PERMANOVA p-values are shown on each panel. (B) Faceted boxplots showing the relative abundance of variables included in the onset XGBoost model, stratified by infectious NF vs Non-infectious NF on the x-axis. Each point represents an individual patient, colored by antibiotic timing. (C) Table reporting unadjusted p-values from univariate tests comparing the relative abundances of these variables between “Before” and “After” onset samples.

Supplemental Table 1: Demographic information for all patients included in this study

Sample ID	Sex	Chemo Intensity	Chemo Type	Complex Karyotype	TET2	RAS	DNMT3	FLT3-ITD	NPM1	ASXL1	IDH2	IDH1	CEBPA	KIT	RUNX1	TP53	NOTCH1	GATA2
0339.2013.001	M	Low	3	0	0	0	0	0	0	0	0	0	1	0	0	1	0	1
0339.2013.002	F	High	2	0	0	1	1	1	0	0	0	0	1	0	0	0	0	1
0339.2013.027	M	High	1	0	1	0	0	1	0	0	0	0	1	0	0	0	0	1
0339.2013.028	M	Low	3	1	0	0	0	1	0	1	0	0	1	0	0	0	0	0
0339.2013.029	M	Low	4	0	1	1	0	0	0	1	0	0	0	0	1	0	0	0
0339.2013.031	M	High	2	0	1	0	0	0	0	0	1	0	0	0	0	0	0	0
0339.2013.033	F	High	2	0	0	1	0	1	0	0	0	0	0	0	0	0	0	0
0339.2013.034	F	High	2	1	1	0	0	0	1	1	1	0	0	0	0	0	0	1
0339.2013.036	M	High	1	0	1	0	0	0	0	0	0	0	1	0	0	0	0	1
0339.2013.037	F	Low	3	0	0	1	0	1	0	0	0	0	0	0	1	0	0	0
0339.2013.038	F	High	1	0	1	0	0	1	0	0	0	0	0	1	0	0	0	0
0339.2013.041	F	Low	3	0	1	0	0	0	0	0	0	0	0	0	0	1	0	0
0339.2013.042	F	High	1	1	1	1	1	0	1	0	0	0	0	0	0	0	0	0
0339.2013.043	F	High	2	0	1	0	1	0	1	0	0	0	0	0	0	0	0	0
0339.2013.045	M	High	1	0	0	0	0	0	0	1	0	0	0	0	0	1	0	0
0339.2013.046	M	Low	3	0	1	1	0	0	0	0	0	0	0	0	0	0	0	0
0339.2013.048	M	Low	3	0	0	0	1	0	0	0	0	0	0	0	0	1	0	0
0339.2013.050	M	High	2	0	0	0	0	0	1	0	0	0	0	0	0	0	1	0
0339.2013.057	M	High	2	0	0	1	1	0	0	0	0	0	0	0	1	0	0	0
0339.2013.058	M	Low	3	0	0	0	0	0	1	0	1	0	0	0	0	0	0	0
0339.2013.059	M	Low	3	0	1	0	0	0	0	0	1	0	0	0	0	0	0	0
0339.2013.060	M	High	2	0	1	0	0	1	1	0	1	0	0	0	0	0	1	0
0339.2013.066	M	Low	3	0	1	1	0	0	0	1	0	0	0	0	0	0	0	0
0339.2013.067	M	High	1	1	0	1	0	0	0	0	0	0	0	0	0	0	0	0
0339.2013.068	M	High	2	0	1	0	0	0	0	0	0	0	1	0	0	0	0	0
0339.2013.069	F	High	1	0	0	1	0	0	1	0	0	1	0	0	0	0	0	0
0339.2013.073	F	Low	3	1	0	1	0	0	0	1	1	0	0	0	0	0	1	0
0339.2013.076	M	Low	4	1	0	1	0	0	0	0	0	0	0	0	0	0	0	0
0339.2013.078	M	High	2	0	0	0	0	1	0	0	0	0	0	0	1	0	0	0
0339.2013.080	M	High	1	0	1	1	0	0	0	0	0	0	0	1	0	0	0	0
0339.2013.081	M	High	2	0	1	0	0	1	0	0	1	1	0	0	0	0	0	0
0339.2013.085	F	Low	3	0	1	0	0	0	0	0	0	0	0	1	0	0	0	0
0339.2013.087	F	High	2	1	0	0	1	1	1	0	0	1	0	0	0	0	0	0
0339.2013.089	F	High	2	1	1	0	1	0	0	1	1	0	1	0	0	0	0	0

Continued: Supplemental Table 1

Sample ID	Sex	Chemo Intensity	Chemo Type	Complex Karyotype	TET2	RAS	DNMT3	FLT3-ITD	NPM1	ASXL1	IDH2	IDH1	CEBPA	KIT	RUNX1	TP53	NOTCH1	GATA2
0339.2013.092	F	High	2	0	1	1	0	1	1	0	0	1	0	0	0	0	0	0
0339.2013.098	M	Low	3	0	0	0	0	0	0	0	0	1	0	0	0	1	0	0
0339.2013.099	F	High	2	0	1	1	0	0	0	0	0	0	0	0	0	0	0	0
0339.2013.102	F	High	1	0	0	0	0	0	0	0	0	0	0	0	0	0	0	0
0339.2013.104	F	High	2	0	1	0	0	1	1	0	0	0	1	0	0	0	0	0
0339-2013-001	M	Low	3	0	0	0	0	0	0	0	0	0	1	0	0	1	0	1
0339-2013-002	F	High	2	0	0	1	1	1	0	0	0	0	1	0	0	0	0	1
0339-2013-003	M	High	2	1	0	0	0	0	0	0	0	1	0	0	0	1	0	0
0339-2013-004	F	High	1	0	0	1	1	0	1	0	0	1	1	0	0	0	0	0
0339-2013-005	F	Low	4	0	1	0	0	0	0	0	1	0	0	0	0	0	0	0
0339-2013-006	M	Low	3	0	0	0	0	1	0	0	0	0	0	0	0	0	0	0
0339-2013-007	F	High	1	0	0	1	1	0	1	0	0	0	0	0	0	0	0	0
0339-2013-008	F	High	1	0	0	0	0	0	0	0	0	0	0	1	0	0	0	0
0339-2013-026	M	High	2	0	1	1	0	1	0	1	0	0	0	0	1	0	0	0
0339-2013-027	M	High	1	0	1	0	0	1	0	0	0	0	1	0	0	0	0	1
0339-2013-028	M	Low	3	1	0	0	0	1	0	1	0	0	1	0	0	0	0	0
0339-2013-031	M	High	2	0	1	0	0	0	0	0	1	0	0	0	0	0	0	0
0339-2013-033	F	High	2	0	0	1	0	1	0	0	0	0	0	0	0	0	0	0
0339-2013-034	F	High	2	1	1	0	0	0	1	1	1	0	0	0	0	0	0	1
0339-2013-036	M	High	1	0	1	0	0	0	0	0	0	0	1	0	0	0	0	1
0339-2013-037	F	Low	3	0	0	1	0	1	0	0	0	0	0	0	1	0	0	0
0339-2013-038	F	High	1	0	1	0	0	1	0	0	0	0	0	1	0	0	0	0
0339-2013-039	F	High	1	0	0	0	0	0	0	0	0	0	0	0	0	0	1	0
0339-2013-041	F	Low	3	0	1	0	0	0	0	0	0	0	0	0	0	1	0	0
0339-2013-043	F	High	2	0	1	0	1	0	1	0	0	0	0	0	0	0	0	0
0339-2013-046	M	Low	3	0	1	1	0	0	0	0	0	0	0	0	0	0	0	0
0339-2013-050	M	High	2	0	0	0	0	0	1	0	0	0	0	0	0	0	1	0
0339-2013-052	F	High	2	0	0	1	0	0	0	1	0	0	0	0	0	0	1	0
0339-2013-055	F	High	2	0	1	0	1	1	1	0	0	0	0	0	0	0	0	0
0339-2013-056	F	Low	3	0	0	0	1	0	0	0	0	0	0	0	0	1	0	0
0339-2013-057	M	High	2	0	0	1	1	0	0	0	0	0	0	0	1	0	0	0
0339-2013-058	M	Low	3	0	0	0	0	0	1	0	1	0	0	0	0	0	0	0
0339-2013-059	M	Low	3	0	1	0	0	0	0	0	1	0	0	0	0	0	0	0
0339-2013-060	M	High	2	0	1	0	0	1	1	0	1	0	0	0	0	0	1	0
0339-2013-062	M	High	2	0	1	0	1	1	1	1	1	0	0	1	0	0	1	0

Continued: Supplemental Table 1

Sample ID	Sex	Chemo Intensity	Chemo Type	Complex Karyotype	TET2	RAS	DNMT3	FLT3-ITD	NPM1	ASXL1	IDH2	IDH1	CEBPA	KIT	RUNX1	TP53	NOTCH1	GATA2
0339-2013-063	F	High	2	1	0	1	1	0	1	0	1	1	0	1	0	1	1	0
0339-2013-065	F	High	2	0	0	1	0	0	0	0	0	0	0	0	0	0	1	0
0339-2013-066	M	Low	3	0	1	1	0	0	0	1	0	0	0	0	0	0	0	0
0339-2013-067	M	High	1	1	0	1	0	0	0	0	0	0	0	0	0	0	0	0
0339-2013-075	M	High	1	0	1	1	0	1	0	0	0	0	0	0	0	0	0	0
0339-2013-076	M	Low	4	1	0	1	0	0	0	0	0	0	0	0	0	0	0	0
0339-2013-077	M	High	1	1	0	0	0	0	0	0	0	0	0	1	0	0	0	0
0339-2013-078	M	High	2	0	0	0	0	1	0	0	0	0	0	0	1	0	0	0
0339-2013-081	M	High	2	0	1	0	0	1	0	0	1	1	0	0	0	0	0	0
0339-2013-085	F	Low	3	0	1	0	0	0	0	0	0	0	0	1	0	0	0	0
0339-2013-087	F	High	2	1	0	0	1	1	1	0	0	1	0	0	0	0	0	0
0339-2013-089	F	High	2	1	1	0	1	0	0	1	1	0	1	0	0	0	0	0
0339-2013-091	M	Low	3	0	1	1	1	1	1	0	0	0	0	0	0	0	0	0
0339-2013-092	F	High	2	0	1	1	0	1	1	0	0	1	0	0	0	0	0	0
0339-2013-094	F	High	2	0	0	1	0	0	0	0	0	0	0	0	0	0	0	0
0339-2013-098	M	Low	3	0	0	0	0	0	0	0	0	1	0	0	0	1	0	0
0339-2013-099	F	High	2	0	1	1	0	0	0	0	0	0	0	0	0	0	0	0
0339-2013-100	M	High	2	0	0	0	0	1	1	0	1	0	0	0	0	0	0	0
15-0780-002	F	High	2	0	0	0	0	0	1	0	0	0	0	0	0	0	0	0
15-0780-003	F	Low	3	0	0	0	1	1	0	0	0	0	0	0	0	0	0	0
15-0780-004	F	High	2	0	1	0	0	0	0	1	1	0	0	0	0	0	0	0
15-0780-006	F	High	2	0	0	0	0	0	0	0	0	0	0	0	0	0	0	0
15-0780-008	F	High	2	0	0	0	1	0	1	1	1	0	0	0	0	0	0	0
15-0780-013	M	Low	3	1	0	1	0	0	0	0	0	0	0	0	0	1	0	0
15-0780-014	M	High	1	0	0	1	0	0	0	1	0	0	0	0	0	0	0	0
15-0780-016	F	High	2	0	0	1	0	0	0	0	0	0	0	0	0	0	0	1
15-0780-017	M	Low	3	0	1	0	1	0	0	0	0	0	0	1	0	0	0	0
15-0780-020	M	High	1	1	1	0	0	0	0	0	0	0	0	0	0	0	0	0
15-0780-021	M	High	1	1	0	0	0	0	0	0	0	0	0	0	0	0	0	0
15-0780-023	M	Low	4	0	0	0	0	0	0	0	0	0	0	0	0	0	0	0
15-0780-024	M	High	2	0	1	1	0	0	0	0	0	0	0	0	1	0	0	1
15-0780-025	M	Low	3	0	1	0	0	0	0	1	1	0	0	0	0	0	0	0
15-0780-026	F	High	1	1	1	0	1	0	0	0	0	0	0	1	0	0	0	0
15-0780-029	F	Low	4	0	0	0	0	0	0	0	0	0	0	0	0	0	0	0
15-0780-030	M	Low	3	0	1	0	1	0	1	0	1	0	0	0	0	0	0	0

Continued: Supplemental Table 1

Sample ID	Sex	Chemo Intensity	Chemo Type	Complex Karyotype	TET2	RAS	DNMT3	FLT3-ITD	NPM1	ASXL1	IDH2	IDH1	CEBPA	KIT	RUNX1	TP53	NOTCH1	GATA2
15-0780-032	F	High	2	1	0	0	0	0	0	0	0	0	0	0	0	1	0	0
15-0780-033	M	Low	4	0	0	0	1	0	0	0	0	0	0	0	0	0	0	0
15-0780-034	M	High	2	0	0	1	1	0	0	0	0	0	0	0	0	0	0	0
15-0780-035	F	High	2	0	0	0	0	1	1	1	0	1	0	0	0	0	0	1
15-0780-036	F	High	1	0	0	0	0	0	0	0	0	0	0	0	0	0	0	0
15-0780-041	F	Low	3	0	1	0	0	0	0	1	0	0	0	0	0	0	0	0
15-0780-043	F	Low	4	0	0	0	0	0	0	0	0	0	0	0	0	0	0	0
15-0780-044	M	Low	4	0	1	0	0	0	0	1	0	0	1	1	0	0	0	1
15-0780-046	F	Low	3	1	1	1	0	0	0	1	0	1	1	0	1	0	1	0
15-0780-051	F	Low	3	0	0	0	1	1	0	1	0	0	0	1	0	0	0	0
15-0780-052	M	High	2	1	1	0	1	1	0	0	1	0	0	0	1	0	0	0
15-0780-055	M	Low	3	0	1	1	1	0	0	0	0	1	0	0	0	0	0	0
15-0780-056	F	Low	3	0	0	1	1	0	0	0	1	0	0	0	0	0	0	0
15-0780-058	F	Low	3	0	0	1	0	0	0	1	0	0	0	0	0	0	0	0
15-0780-059	F	Low	3	0	0	0	1	0	0	1	0	0	0	0	0	0	0	0
15-0780-060	M	Low	3	0	1	0	0	0	0	0	0	0	0	0	1	0	0	0
15-0780-061	M	Low	3	1	1	0	0	0	0	0	0	0	0	0	0	1	0	0
15-0780-062	F	High	1	1	1	0	0	0	0	0	0	0	0	0	0	1	1	0
15-0780-063	F	Low	4	0	1	0	1	0	0	0	0	0	1	0	0	0	0	0
15-0870-033	M	Low	4	0	0	0	1	0	0	0	0	0	0	0	0	0	0	0
15-0870-034	M	High	2	0	0	1	1	0	0	0	0	0	0	0	0	0	0	0
15-0870-035	F	High	2	0	0	0	0	1	1	1	0	1	0	0	0	0	0	1
15-0870-040	M	Low	4	0	1	0	0	0	0	0	0	0	0	0	1	1	0	0

Supplemental Table 2: Summary of patient overlap between baseline and fever-onset cohorts.

Fever Group	Total Patients (N)	Unique to Baseline (N)	Unique to Onset (N)	Overlapping Patients (N)
Infectious NF	49	6	16	27
Non-Infectious NF	18	1	4	13
No fever	28	14	5	9
Total	95	21	25	49

Supplemental Table 3: The optimization search spaces for the baseline and onset models

Hyperparameter	Default	Baseline-A	Baseline-B	Baseline-C	Baseline-D	Onset
gamma	0	0	0	0	0	0
eta	0-1	0-1	0-1	0.1-1	0-0.3	0.1-1
max _depth	1-50	6	6	6	6	6
colsample_bytree	0-1	0-0.5	0-1	0-0.6	0-0.8	0-0.8
min_child_weight	1-50	1-6	1-6	1-8	1-6	1-4
subsample	0-1	0.1-0.6	0.4-0.8	0.25-1	0.6-0.9	0.5-1
nrounds	100-1000	100-1000	100-1000	100-1000	100-1000	100-1000

Baseline models: A: all variables, B: CLR-transformed variables, C: Univariate screened, D: CLR-transformed Univariate screened variables.

Supplemental Table 4: Characteristics of patients included in baseline analyses.

Patient Characteristics	Infectious NF	Non-Infectious NF	No Fever	P-Value[2]	Test-Statistic
Patient Count, N(%)	33	14	23		
Sex, N(%)				0.243	2.833
Female	18	4	12		
Male	15	10	11		
Days to any NF, mean (range)	16.4(4-53)	13.4(6-31)	--		
Days to Infection, mean (range)	18.6(4-53)	--	--		
Chemotherapy Intensity, N(%)				0.271	2.614
High	21	9	10		
Low	12	5	13		
Chemotherapy Type, N(%)				0.384	6.364
Fludarabine	7	4	4		
Non-Fludarabine(High)	14	5	6		
Hypomethylators	11	3	8		
Other(Low)	1	2	5		
Antibiotic Administration[1], N(%)					
Pip-Tazo	5	1	3	0.754	0.564
Cephalosporin	13	3	9	0.459	1.556
Carbapenem	8	1	2	0.179	3.444
AML Somatic Mutations, N(%)					
TET2	13	5	13	0.344	2.133
RAS	11	4	8	0.924	0.159
DNMT3	9	4	4	0.639	0.895
FLT3-ITD	10	3	4	0.520	1.307
NPM1	8	3	2	0.323	2.261
ASXL1	7	3	5	0.999	0.002
IDH2	7	4	1	0.115	4.323
IDH1	5	0	1	0.161	3.659
CEBPA	2	3	5	0.178	3.450
KIT	1	1	4	0.164	3.612
RUNX1	2	1	3	0.641	0.889
TP53	4	2	3	0.979	0.042
NOTCH1	0	2	2	0.117	4.289
GATA2	5	2	2	0.765	0.536
Complex Karyotype, N(%)	7	3	5	0.999	0.002

[1] Antibiotic Administration defined as administered for at least 72 consecutive hours

[2] P-value and X2 value was determined via Chi-Squared Test using R

Abbreviation: NF, Neutropenic Fever;

Supplemental Table 5: Comparison of key clinical characteristics between overlapping and unique cohort participants

Patient Characteristics	Shared	Baseline-Only	Onset-Only	P-value[1]	Test-Statistic
Patient Count, N	49	21	25		
Sex, N				0.812	0.418
Female	24	10	14		
Male	25	11	11		
Days to NF, median (range)	16.3(4-53)	10.3(6-14)	15 (0-135)		
Days to Infection, median (range)	19.6 (4-53)	13.4 (6-19)	18 (0-135)		
Chemotherapy Intensity, N				0.726	0.639
High	27	13	16		
Low	22	8	9		
Chemotherapy Type, N				0.168	9
Fludarabine	6	9	6		
Non-Fludarabine (High)	21	4	10		
Hypomethylators	16	6	7		
Other (Low)	6	2	2		
Antibiotic Administration[2], N					
Pip-Tazo	6	3	6	0.414	1.766
Cephalosporin	15	10	16	0.0208	7.741
Carbapenem	9	2	14	0.000334	16.011
AML Somatic Mutations, N					
TET2	22	9	12	0.938	0.127
RAS	16	7	10	0.812	0.418
DNMT3	12	5	9	0.529	1.275
FLT3-ITD	14	3	6	0.441	1.636
NPM1	10	3	8	0.325	2.249
ASXL1	10	5	6	0.919	0.170
IDH2	11	1	5	0.199	3.232
IDH1	5	1	5	0.250	2.775
CEBPA	8	2	2	0.528	1.276
KIT	3	3	5	0.191	3.307
RUNX1	4	2	3	0.867	0.284
TP53	4	5	4	0.202	3.201
NOTCH1	2	2	6	0.0302	7.002
GATA2	8	1	0	0.0539	5.843
Complex Karyotype, N	9	6	5	0.624	0.943

1. P-value and Test statistics were determined via Chi-Squared Test using R
2. Antibiotic administration was defined as administered for at least 72 consecutive hours

Supplemental Table 6: Comparison of baseline class abundances across fever outcome groups.

		Dunn's Test for Multiple Comparisons p-value		
Class	Kruskal-Wallis pvalue	Infectious NF – No Fever	Infectious NF – Non-Infectious NF	No Fever – Non-Infectious NF
Actinobacteria	0.252	0.112	0.313	0.747
Alphaproteobacteria	0.867	0.622	0.713	0.961
Anaerolineae	0.135	1	0.061	0.078
Bacilli	0.816	0.527	0.879	0.716
Bacteroidia	0.435	0.642	0.334	0.200
Blastocatellia	0.444	0.512	0.426	0.203
Campylobacteria	0.502	0.526	0.482	0.242
Clostridia	0.587	0.302	0.749	0.599
Coriobacteriia	0.754	0.641	0.475	0.766
Cyanobacteriia	0.946	0.743	0.864	0.919
Deinococci	0.388	0.289	0.651	0.202
Desulfovibrionia	0.516	0.664	0.250	0.463
Fusobacteriia	0.317	0.207	0.220	0.886
Gammaproteobacteria	0.763	0.513	0.585	0.991
Methanobacteria	0.363	0.157	0.527	0.590
Negativicutes	0.256	0.732	0.102	0.207
Synergistia	0.620	0.331	0.803	0.586
Thermoplasmata	0.444	0.512	0.426	0.203
Vampirivibrionia	0.841	0.824	0.557	0.708
Verrucomicrobiae	0.305	0.372	0.136	0.492

Abbreviations: NF, Neutropenic Fever

Supplemental Table 7: Comparison of class abundances at fever onset across fever outcome groups.

Class	Kruskal-Wallis pvalue	Dunn's Test for Multiple Comparisons p-value		
		Infectious NF – No Fever	Infectious NF – Non-Infectious NF	No Fever – Non-Infectious NF
Actinobacteria	0.343	0.156	1	0.243
Alphaproteobacteria	0.061	0.018	0.727	0.097
Bacilli	0.378	0.565	0.170	0.530
Bacteroidia	0.513	0.686	0.341	0.273
Blastocatellia	0.135	0.422	0.114	0.054
Campylobacteria	0.029	0.031	0.325	0.010
Clostridia	0.145	0.050	0.713	0.186
Coriobacteriia	0.021	0.006	0.665	0.053
Cyanobacteriia	0.120	0.044	0.364	0.352
Deinococci	0.370	0.273	0.260	1.000
Desulfovibrionia	0.780	0.800	0.485	0.725
Fusobacteriia	0.320	0.902	0.153	0.212
Gammaproteobacteria	0.804	0.591	0.615	0.969
Methanobacteria	0.369	0.551	0.166	0.535
Negativicutes	0.434	0.313	0.303	0.996
Synergistia	0.064	0.029	0.174	0.479
Thermoplasmata	0.111	0.722	0.055	0.067
Vampirivibrionia	0.509	0.661	0.348	0.265
Verrucomicrobiae	0.703	0.670	0.421	0.769
Anaerolineae	Na	Na	Na	Na

Abbreviations: NF, Neutropenic Fever

Supplemental Table 8: Baseline genera differences between infectious NF and non-infectious NF, Mann-Whitney p≤0.3

Group	log2FC	pvalue	Adjusted pvalue
Haemophilus	0.035091	0.004134	0.381316
UCG-010_ge (O: Oscillospirales)	-4.41076	0.004534	0.381316
Allisonella	-9.6309	0.008554	0.381316
Lachnospiraceae_FCS020_group	-2.15724	0.008972	0.381316
Lachnospira	-0.92095	0.021939	0.745942
Coprococcus	-2.1977	0.039489	0.771283
Prevotella	-3.66006	0.054399	0.771283
Enterobacter	3.195826	0.063617	0.771283
Weissella	0.939158	0.067376	0.771283
Fusicatenibacter	-0.56026	0.071129	0.771283
Erysipelotrichaceae_ge	4.404226	0.076884	0.771283
GCA-900066575 (O: Lachnospirales)	-5.26972	0.081292	0.771283
Lachnospiraceae_NK4A136_group	-0.52982	0.092391	0.771283
Adlercreutzia	5.342827	0.092432	0.771283
uncultured_ge	-4.53654	0.106008	0.771283
Gordonibacter	3.591903	0.110397	0.771283
UCG-003 (O: Oscillospirales)	-0.84554	0.119646	0.771283
Dubosiella	2.775883	0.12263	0.771283
Staphylococcus	-7.85581	0.124215	0.771283
GCA-900066755 (O: Lachnospirales)	2.662685	0.130706	0.771283
DNF00809 (O: Coriobacteriales)	4.54053	0.130706	0.771283
Dorea	-0.33226	0.133187	0.771283
Escherichia-Shigella	0.26096	0.133187	0.771283
Intestinibacter	1.875288	0.136192	0.771283
Granulicatella	-2.47451	0.136484	0.771283
Akkermansia	0.737659	0.137745	0.771283
Rothia	-2.49117	0.140646	0.771283
Lachnospiraceae_ge	-0.44933	0.145746	0.771283
Oribacterium	-7.15261	0.150215	0.771283
Collinsella	0.546053	0.16109	0.771283
Eggerthellaceae_unclass	0.646526	0.1643	0.771283
Roseburia	-0.59986	0.167877	0.773835
Lachnospiraceae_UCG-004	-1.47821	0.182796	0.773835
Dialister	1.751702	0.199335	0.792752
Barnesiella	-2.14982	0.209917	0.792752
Lachnospiraceae_ND3007_group	0.830295	0.232104	0.792752
Muribaculaceae_ge	-0.99371	0.23536	0.839872
Acidaminococcus	0.3255	0.246146	0.89176
Gemella	-2.21303	0.257645	0.915021
Lachnospiraceae_unclass	-0.13546	0.278221	0.926546
Flavonifractor	1.250282	0.281933	0.926546
Phascolarctobacterium	-0.07828	0.284141	0.926546
Fusobacterium	4.097308	0.294641	0.926546

Supplemental Table 9: Baseline genera differences between infectious NF and no fever, Mann-Whitney p≤0.3

Group	log2FC	pvalue	Adjusted pvalue
Anaerococcus	-4.35158	0.01075	0.910236
Enterobacter	-1.99326	0.014056	0.910236
Lactonifactor	-2.05718	0.040355	0.910236
Escherichia-Shigella	1.045434	0.05278	0.910236
Coprococcus	-1.60294	0.053018	0.910236
Pseudomonas	-3.90711	0.054635	0.910236
Prevotella	-1.37905	0.059137	0.910236
Allisonella	-5.23288	0.059377	0.910236
Barnesiella	-0.30742	0.061547	0.910236
Incertae_Sedis	-1.20908	0.080235	0.910236
Family_XIII_AD3011_group (O: Peptostreptococcales-Tissierellales)	-0.53198	0.084307	0.910236
Actinomyces	-3.44267	0.087372	0.910236
Weissella	6.496118	0.097601	0.910236
Fournierella	2.735459	0.099494	0.910236
Eubacterium	-1.36087	0.102555	0.910236
Clostridia_UCG-014_ge	-4.54765	0.105934	0.910236
Bacillus	-3.46886	0.106351	0.910236
Methanobrevibacter	-1.26628	0.125722	0.910236
Sutterella	-5.15207	0.136609	0.910236
Tyzzerella	-2.3279	0.138059	0.910236
Ruminiclostridium	-3.15667	0.144521	0.910236
Shuttleworthia	2.643007	0.146237	0.910236
Christensenellaceae_R-7_group	-1.02437	0.14755	0.910236
Lachnospiraceae_unclass	-0.2137	0.151816	0.910236
Fusicatenibacter	-0.78346	0.155037	0.910236
Alloprevotella	-0.2269	0.155523	0.910236
Raoultella	-3.89452	0.155523	0.910236
Dorea	0.056827	0.155927	0.910236
Rothia	-2.06082	0.16217	0.910236
Hungatella	0.767753	0.168088	0.910236
uncultured_ge	-3.12597	0.182058	0.910236
Moryella	-2.05542	0.184727	0.910236
UCG-010_ge (O: Oscillospirales)	-2.98353	0.185909	0.910236
Abiotrophia	-9.74249	0.187402	0.910236
Phascolarctobacterium	-1.56011	0.207151	0.910236
Leuconostoc	-2.42637	0.220782	0.978215
Agathobacter	-1.00401	0.236056	0.985588
Acinetobacter	2.02891	0.245913	0.985588
Caproiciproducens	3.985494	0.26942	0.985588
Scardovia	-3.17408	0.26942	0.985588
Flavonifractor	-0.24536	0.281821	0.985588
Subdoligranulum	-1.34793	0.284656	0.985588
UCG-009 (O: Oscillospirales)	-0.82325	0.285407	0.985588
UCG-002 (O: Oscillospirales)	-0.91458	0.293856	0.985588
Rikenellaceae_RC9_gut_group	5.124013	0.296625	0.985588

Supplemental Table 10: Baseline genera differences between non-infectious NF and no fever, Mann-Whitney p≤0.3

Group	log2FC	pvalue	Adjusted pvalue
Lachnospiraceae_FCS020_group	1.81797	0.014321	0.961739
Haemophilus	1.365191	0.020511	0.961739
Adlercreutzia	-7.68237	0.031707	0.961739
Flavonifractor	-1.49565	0.042353	0.961739
Alloprevotella	6.036975	0.042501	0.961739
Lachnospira	-1.4761	0.052495	0.961739
Pseudomonas	-1.57267	0.064177	0.961739
UC5-1-2E3 (O: Lachnospirales)	2.730879	0.064327	0.961739
Hungatella	1.623126	0.077926	0.961739
Sutterella	-11.3599	0.085887	0.961739
Tyzzerella	-2.00956	0.089706	0.961739
Staphylococcus	4.151191	0.097947	0.961739
Gordonibacter	-2.63791	0.107847	0.961739
DNF00809 (O: Coriobacteriales)	-5.40068	0.112499	0.961739
GCA-900066755 (O: Lachnospirales)	-3.56802	0.112499	0.961739
Gemella	2.002208	0.11771	0.961739
UCG-010_ge (O: Oscillospirales)	1.427235	0.11816	0.961739
Leuconostoc	0.41277	0.12109	0.961739
Intestinibacter	0.476613	0.126855	0.961739
Acidaminococcus	3.643315	0.133589	0.961739
Lachnospiraceae_ND3007_group	-0.58664	0.135775	0.961739
Anaerococcus	-3.91747	0.155504	0.961739
Fournierella	0.320455	0.177725	0.961739
Lachnospiraceae_NK4A136_group	2.6116	0.192246	0.961739
Erysipelotrichaceae_ge	-4.08782	0.20001	1
GCA-900066575 (O: Lachnospirales)	4.488686	0.206023	1
Lachnospiraceae_XPB1014_group	-0.80321	0.212093	1
Subdoligranulum	-2.98264	0.214561	1
Butyricimonas	0.889327	0.221981	1
Christensenellaceae_R-7_group	-0.59852	0.224385	1
Corynebacterium	1.082572	0.237509	1
Oribacterium	6.255491	0.242662	1
Dubosiella	-2.1618	0.251596	1
Hydrogenoanaerobacterium	1.012338	0.261633	1
Lactococcus	-1.18272	0.263863	1
UCG-003 (O: Oscillospirales)	1.741044	0.273265	1
Allisonella	4.398019	0.286746	1
Lactobacillus	0.139851	0.286958	1
Holdemanella	-0.30561	0.29422	1
CAG-56 (O: Lachnospirales)	-2.65529	0.29422	1

Supplemental Table 11: Metabolite levels for patients included in baseline analyses.

Sample ID	2HG	Acetic Acid	Buty-rate	Glyceric Acid	Keto-leucine	Lactate	Malate	Pentanoic Acid	Pro-pionate	Suc-cinate
0339.2013.006	281.5	1357.6	67.6	24.3	97.9	23.8	200.3	25.0	546.9	195.2
0339.2013.007	51.8	1891.6	31.1	9.3	60.5	21.7	20.4	2.5	41.4	348.0
15-0780-004	8.9	1875.4	72.1	33.3	13.0	8.3	118.3	15.6	235.5	607.2
15-0780-006	21.1	1628.8	79.9	46.9	13.0	9.2	66.7	2.0	802.3	390.6
15-0780-008	216.1	3139.0	47.6	19.3	30.3	1017.1	101.3	0.3	557.4	1710.1
15-0780-013	22.2	2467.4	97.8	31.4	97.6	14.1	67.7	23.2	650.6	46.2
15-0780-014	10.7	1586.4	78.9	29.3	3.5	64.1	106.1	4.1	498.7	1045.9
15-0780-016	20.3	660.1	0.2	66.7	2.0	20.1	124.9	0.2	25.2	10.6
15-0780-020	4.3	1554.2	0.1	16.1	21.3	226.0	43.4	0.0	4.1	67.9
15-0780-021	40.0	2974.3	11.7	41.9	107.5	71.0	93.3	2.0	393.8	1735.7
15-0780-029	15.8	1392.3	156.8	35.5	42.3	15.8	129.3	52.0	1412.7	68.9
15-0780-035	59.4	1916.7	0.5	41.1	2.6	223.2	246.8	0.3	98.1	869.3
15-0780-036	26.9	1431.6	36.1	31.3	45.2	39.3	70.6	20.9	1633.7	52.6
15-0780-051	10.1	1514.0	288.1	21.9	10.1	8.0	76.4	42.0	1781.5	146.5
15-0780-052	6.5	838.9	155.6	34.5	2.5	6.5	78.2	33.0	1696.4	261.9
15-0780-056	19.2	1881.9	98.0	34.1	51.8	64.1	136.1	29.8	1121.4	212.0
0339.2013.041	11.9	1729.6	106.6	23.6	22.5	9.9	37.8	16.7	235.4	904.5
0339.2013.042	2.8	310.5	10.2	2.2	11.5	1.8	6.1	4.6	42.2	4.0
0339.2013.043	37.7	1283.2	51.8	38.5	26.3	12.2	14.1	1.9	326.7	126.7
0339.2013.046	25.8	1211.9	67.6	11.7	24.9	28.2	16.7	1.8	163.4	8.2
0339.2013.002	16.5	1893.1	145.4	38.1	43.0	12.3	79.5	26.7	557.2	163.5
0339.2013.031	19.0	857.0	41.6	40.0	164.7	10.2	57.1	19.8	235.6	82.0
0339.2013.034	22.3	2720.9	167.5	36.9	20.7	23.9	84.7	59.9	1202.0	322.5
0339.2013.036	54.6	660.3	0.4	19.9	7.2	61.8	90.4	0.0	13.4	51.5
0339.2013.037	80.9	1670.5	256.5	54.8	41.4	91.9	58.5	6.8	1279.6	48.9
0339.2013.048	116.7	2740.3	50.8	31.6	85.5	65.3	219.1	6.2	157.8	736.7
0339.2013.066	25.6	2764.5	60.6	33.2	96.3	4.8	32.3	32.2	858.7	74.1
0339.2013.078	96.4	3071.7	2.8	60.9	42.8	65.9	189.3	0.2	593.9	1461.6
0339.2013.081	11.3	1061.5	21.4	14.4	66.8	8.5	57.6	11.0	105.5	50.6
0339.2013.087	53.9	3797.7	81.6	19.1	102.5	462.2	229.6	3.4	748.1	526.3
0339.2013.092	33.9	1647.7	243.5	61.7	122.4	33.9	101.7	37.9	1422.8	228.6
0339.2013.098	20.0	2049.7	10.8	37.2	44.8	9.7	33.0	15.2	199.4	25.5
0339.2013.059	55.2	2160.0	95.2	31.2	63.5	11.0	144.8	25.5	739.8	108.1
15-0780-003	168.0	1155.6	55.1	34.0	10.1	45.8	93.6	1.6	409.8	1568.7
15-0780-017	27.5	757.5	0.2	18.9	3.5	31.3	97.1	0.1	2.3	22.5
15-0780-023	43.8	1074.3	0.9	24.8	9.5	17.2	189.8	0.1	5.8	52.2
15-0780-024	20.6	1810.6	51.6	20.6	71.8	10.8	92.9	20.5	206.4	121.0
15-0780-032	59.9	1399.2	20.1	30.5	10.8	13.6	128.3	1.0	139.2	877.1
15-0780-033	157.2	3103.8	61.5	83.4	15.5	15.8	159.4	95.6	2108.4	34.6

Continued: Supplemental Table 11

Sample ID	2HG	Acetic Acid	Buty-rate	Glyceric Acid	Keto-leucine	Lactate	Malate	Pentanoic Acid	Pro-pionate	Suc-cinate
15-0780-044	35.4	1847.5	136.8	46.5	22.8	14.5	155.5	40.5	417.1	87.9
15-0780-058	7.0	1223.4	267.7	26.9	0.8	12.9	36.9	33.4	1145.1	1095.7
15-0780-060	38.5	1526.8	166.1	41.7	118.6	27.6	92.5	45.7	1118.0	634.3
15-0780-061	22.0	1187.7	139.0	19.0	67.0	7.9	23.5	36.4	408.5	32.8
15-0780-062	48.4	1550.1	177.0	16.7	1.8	27.7	14.8	4.4	434.1	1150.5
15-0780-063	16.5	1507.9	149.5	27.3	65.8	10.7	57.8	4.2	1265.5	153.7
0339.2013.028	12.3	379.1	0.2	18.2	3.7	29.3	132.6	0.0	3.5	15.1
0339.2013.029	67.6	2431.1	98.6	21.9	120.2	22.8	91.2	28.6	360.1	229.9
0339.2013.033	11.6	1234.6	36.1	23.4	61.9	9.9	135.9	20.7	397.3	64.4
0339.2013.068	31.5	4082.2	216.9	37.9	43.2	13.7	41.4	50.4	818.3	49.5
0339.2013.069	16.0	2397.8	35.4	53.4	86.7	19.6	55.3	17.4	377.1	67.9
0339.2013.073	51.2	1673.5	0.4	30.1	2.9	26.8	108.5	0.1	4.4	525.1
0339.2013.080	39.4	2157.6	312.2	94.5	88.0	42.1	115.9	37.4	1430.8	153.8
0339.2013.085	130.0	1693.5	38.5	83.3	225.9	68.2	180.0	24.8	1058.3	219.9
0339.2013.099	109.4	1914.1	23.9	40.5	73.1	18.5	137.0	1.1	88.6	218.0
0339.2013.102	3491.9	0.0	10.7	0.0	188.2	4337.0	3998.6	7.0	4881.6	26248.3
0339.2013.104	36.3	3052.0	133.9	51.7	27.3	8.5	96.9	28.8	326.2	271.0
0339.2013.005	75.7	2069.7	112.1	58.0	245.1	21.9	137.6	26.5	867.1	75.4
15-0780-034	23.1	2455.2	87.9	36.7	135.6	24.5	117.4	2.9	660.1	344.5
15-0780-059	18.7	858.2	126.2	15.0	4.7	8.5	9.2	51.9	605.3	491.8
0339.2013.045	171.5	3015.7	73.0	20.7	601.2	515.9	31.0	2.5	198.6	156.0
0339.2013.001	30.9	1628.5	206.8	44.3	51.0	14.8	75.6	18.4	899.2	222.7
0339.2013.027	14.1	866.6	0.1	26.8	3.4	26.2	199.9	0.0	6.5	14.4
0339.2013.038	88.4	939.3	2.2	14.0	22.5	81.1	55.1	0.1	75.2	58.8
0339.2013.050	145.5	3201.6	130.1	37.2	115.9	185.5	114.8	37.5	799.1	199.8
0339.2013.067	69.5	1841.4	56.9	32.8	41.4	19.9	41.5	23.9	418.2	189.8
0339.2013.076	23.9	2087.3	149.1	37.2	74.8	13.7	90.8	39.3	618.0	50.7
0339.2013.089	17.0	2403.9	240.3	69.7	133.6	20.3	150.0	73.4	1793.1	70.9
0339.2013.057	18.1	2056.6	23.9	12.9	19.6	5.9	17.6	11.9	168.5	48.4
0339.2013.058	254.2	3453.8	23.4	63.4	35.8	323.8	317.0	0.8	336.2	1251.4
0339.2013.060	81.1	3526.8	294.9	58.6	33.1	9.3	128.7	12.2	1309.6	65.1

Supplemental Table 12: Variable selection model descriptions and variables at baseline.

Model	Description	Variables Included		
		Microbial	Metabolite	Clinical
A	All raw variables	170	10	23
B	All variables, CLR-transformed microbial variables	170	10	23
C	Variables chosen through univariate screening (p<0.3)	47	3	6
D	Variables chosen through univariate screening (p<0.3), CLR-transformed	47	3	6
Onset	Variables chosen through univariate screening (p<0.3), CLR-transformed microbial variables	54	6	7

Abbreviations: CLR, center log ratio

Supplemental Table 13: Feature importance scores derived from XGBoost models

Variable	ModelA	ModelB	ModelC	ModelD	Onset
AceticAcid	0.1329	0.1410	0.1086	0.1039	
Akkermansia	0.1938	0.2128	0.1723	0.1676	
Batch					0.0547
Butyrate					0.1151
Collinsella			0.0698	0.0842	
Coprococcus	0.0907	0.0834	0.0711	0.0736	
DTU089					0.0737
Eisenbergiella					0.0829
Enterobacter	0.0650	0.0720	0.0564	0.0582	
Enterococcus					0.1225
Escherichia_Shigella	0.0932	0.0885	0.0938	0.0777	
Flavonifractor	0.0987	0.0994	0.1030	0.1093	
Fusicatenibacter	0.0525	0.0498			
GCA_900066575			0.0534	0.0678	
Gemella					0.0994
Granulicatella			0.0535		
Lachnospiraceae_FCS020_group	0.1891	0.2099	0.1452	0.1587	
Lachnospiraceae_ge	0.0260	0.0332			
Lachnospiraceae_NK4A136_group					0.1918
Prevotella					0.0672
Roseburia					0.0923
Rothia	0.0581		0.0730	0.0990	
Ruminococcaceae_unclass					0.1003

Supplemental Table 14: Genera differences at fever onset between infectious NF and non-infectious NF, Mann-Whitney p≤0.3

Group	log2FC	pvalue	Adjusted pvalue
GCA-900066575 (O: Lachnospirales)	-3.0104	0.001285	0.072035
Lachnospiraceae_NK4A136_group	-0.37852	0.001412	0.072035
Roseburia	-2.20162	0.001983	0.072035
DTU089 (O: Oscillospirales)	0.235495	0.004031	0.109845
Lachnospiraceae_ND3007_group	-0.413	0.005341	0.111174
Gemella	-2.052	0.007269	0.111174
Marvinbryantia	-1.47815	0.00816	0.111174
UC5-1-2E3 (O: Lachnospirales)	-2.80531	0.01317	0.111174
Coprococcus	-0.8354	0.013473	0.136297
Ruminococcaceae_unclass	0.423787	0.013755	0.136297
Enterococcus	6.435295	0.015624	0.136297
Lachnospiraceae_FCS020_group	-1.67147	0.019528	0.141917
Incertae_Sedis	-1.10776	0.019618	0.152744
Clostridia_UCG-014_ge	-0.79765	0.02478	0.152744
Faecalibacterium	0.451434	0.029174	0.180071
Agathobacter	-1.71273	0.036664	0.19875
Dorea	-0.79643	0.041526	0.235082
Eisenbergiella	0.137366	0.050859	0.251465
Oscillibacter	0.059526	0.055537	0.282837
Butyricicoccus	0.458152	0.05625	0.282837
Collinsella	1.036528	0.057086	0.282837
Clostridia_vadinBB60_group_ge	5.761225	0.061575	0.282837
UCG-005 (O: Oscillospirales)	-2.01356	0.064502	0.29181
Barnesiella	-2.39037	0.068545	0.292945
Paraprevotella	-1.07782	0.075576	0.298854
Lactobacillus	-0.64888	0.075743	0.303684
Oscillospiraceae_unclass	-0.60699	0.078011	0.303684
Anaerofilum	2.879699	0.092609	0.303684
Prevotella	-1.9064	0.094854	0.344637
Colidextribacter	-1.26949	0.106299	0.344637
Lachnospiraceae_ge	-0.51492	0.107473	0.36608
Lachnospiraceae_unclass	0.744867	0.116787	0.36608
Parabacteroides	-0.3329	0.122523	0.385752
Faecalitalea	-0.82636	0.140056	0.392793
UCG-002 (O: Oscillospirales)	-1.64454	0.146146	0.430537
Ruminococcaceae_ge	-1.84194	0.150792	0.430537
Moryella	1.033479	0.172758	0.430537
Methanobrevibacter	-0.52165	0.175805	0.432536
Ruminococcus	-1.05868	0.17872	0.474417
Escherichia-Shigella	0.184641	0.182803	0.474417
Lachnospira	0.887544	0.195285	0.474417
Hungatella	-0.45558	0.211223	0.474417
Monoglobus	0.323479	0.211781	0.495024
Terrisporobacter	2.636285	0.215134	0.504309
Desulfovibrio	-1.25261	0.220627	0.504309
Negativibacillus	-0.51393	0.22796	0.504309
Alistipes	-0.94095	0.233448	0.504309
Oscillospirales_ge	-1.80213	0.235413	0.504309

Continued: Supplemental Table 14

Group	log2FC	pvalue	Adjusted pvalue
Anaerostipes	0.71611	0.238119	0.504309
CAG-352 (O: Lachnospirales)	-0.97589	0.240588	0.504309
Sellimonas	1.650552	0.249679	0.51349
Adlercreutzia	4.66587	0.286458	0.573946
Atopobium	5.408615	0.292341	0.573946
Fusicatenibacter	0.64063	0.294871	0.573946

Supplemental Table 15: Genera differences at fever onset between infectious NF and no fever, Mann-Whitney p≤0.3

Group	log2FC	pvalue	Adjusted pvalue
Paraprevotella	-3.39467	0.000592	0.04176
Adlercreutzia	-2.29417	0.000766	0.04176
Butyricicoccus	-1.39844	0.001921	0.057499
Eggerthella	-1.91262	0.002605	0.057499
GCA-900066575 (O: Lachnospirales)	-3.10589	0.002638	0.057499
Ruminococcaceae_ge	-5.09737	0.003745	0.068033
Marvinbryantia	-1.5362	0.004797	0.074694
Lachnospiraceae_ND3007_group	-3.67688	0.007187	0.087875
Sellimonas	-1.21962	0.007262	0.087875
Tyzzerella	-0.58187	0.008443	0.087875
Moryella	-1.66742	0.008868	0.087875
DTU089 (O: Oscillospirales)	0.624083	0.011115	0.100963
UC5-1-2E3 (O: Oscillospirales)	-0.9702	0.013203	0.105213
Coriobacteriales_Incertae_Sedis_unclass	-0.04742	0.013564	0.105213
Roseburia	-1.72997	0.014479	0.105213
Gordonibacter	2.322128	0.016656	0.113466
Collinsella	-0.86222	0.019572	0.119064
Coprococcus	-1.83599	0.019662	0.119064
Subdoligranulum	-1.30318	0.022557	0.129408
Lachnospiraceae_NK4A136_group	-1.37241	0.024437	0.131394
CAG-56 (O: Lachnospirales)	-5.06056	0.025314	0.131394
Lachnospiraceae_FCS020_group	-2.40277	0.027338	0.135446
Incertae_Sedis	-1.69797	0.032075	0.152006
Oscillibacter	0.933846	0.038762	0.16852
Escherichia-Shigella	3.077423	0.040543	0.16852
Lachnospiraceae_unclass	-0.10822	0.042901	0.16852
Lachnospiraceae_ge	-1.30737	0.043883	0.16852
Fournierella	2.296268	0.044006	0.16852
Flavonifractor	0.088895	0.047348	0.16852
Chloroplast_ge	6.702715	0.054048	0.172029
Lactobacillus	-0.12177	0.063745	0.190039
Solobacterium	3.679435	0.070372	0.198101
Enterococcus	0.114757	0.071439	0.210551
Oscillospiraceae_unclass	-1.08651	0.079391	0.222482
Monoglobus	0.650952	0.079681	0.222482
Colidextribacter	1.16771	0.086242	0.234735
Acinetobacter	7.753695	0.092183	0.234735
Agathobacter	-1.80124	0.093686	0.247378
Gemella	-1.52443	0.094099	0.250165
Fusicatenibacter	-1.61075	0.097429	0.250165
Stenotrophomonas	11.02883	0.099816	0.250165
Blautia	-0.59962	0.102758	0.252851
Oscillospirales_ge	-0.4204	0.104824	0.253022
Bilophila	-0.70645	0.107612	0.253907

Continued: Supplemental Table 15

Group	log2FC	pvalue	Adjusted pvalue
Clostridia_vadinBB60_group_ge	6.59279	0.109663	0.254325
Sutterella	-3.66788	0.112185	0.254754
UCG-002 (O: Oscillospirales)	-0.3941	0.118908	0.264509
Bifidobacterium	-2.51818	0.127546	0.278051
Weissella	9.001369	0.142028	0.30355
Staphylococcus	-1.7471	0.157653	0.330464
Eisenbergiella	0.322617	0.174328	0.352809
UCG-005 (O: Oscillospirales)	0.317512	0.174786	0.352809
Dorea	-1.57512	0.183818	0.364256
Hungatella	2.628418	0.187141	0.364256
Lactococcus	4.610578	0.191062	0.365363
Negativibacillus	0.738462	0.205285	0.385795
Lachnospiraceae_UCG-006	8.946769	0.214869	0.396961
Ruminococcaceae_unclass	0.782644	0.222328	0.403896
Barnesiella	1.760814	0.235713	0.421193
Faecalibacterium	-0.66173	0.260446	0.45788
Alloprevotella	8.058084	0.278843	0.482443

Supplemental Table 16: Genera differences at fever onset between non-infectious NF and no fever, Mann-Whitney p≤0.3

Group	log2FC	pvalue	Adjusted pvalue
Adlercreutzia	-6.96004	0.019977	0.964076
Gordonibacter	-4.05104	0.063484	0.964076
Mitochondria_ge	3.174266	0.080004	0.964076
Eggerthella	-2.93213	0.089599	0.964076
Flavonifractor	-0.9888	0.118726	0.964076
CAG-56 (O: Lachnospirales)	-5.53593	0.119295	0.964076
Sellimonas	-2.87017	0.121566	0.964076
Atopobium	-1.05591	0.128977	0.964076
Coriobacteriales_Incertae_Sedis_unclass	-1.94617	0.133468	0.964076
Butyricicoccus	-1.8566	0.16736	0.964076
Clostridia_UCG-014_ge	-1.17188	0.172809	0.964076
Solobacterium	4.624884	0.181266	0.964076
Tyzzerella	0.536045	0.192352	0.964076
Erysipelotrichaceae_ge	-1.28534	0.204953	0.964076
Leuconostoc	-1.74316	0.211127	0.964076
Subdoligranulum	-2.02769	0.228047	0.964076
Ruminococcaceae_unclass	0.358857	0.244175	0.964076
Parasutterella	-5.49822	0.254219	0.964076
Fusobacterium	0.044605	0.257033	0.964076
Anaerofilum	1.263573	0.263699	0.964076
Chloroplast_ge	0.322619	0.265296	0.964076
Paraprevotella	-2.31685	0.271151	0.964076
Staphylococcus	-6.46722	0.277298	0.964076
Blautia	-0.97402	0.289776	0.964076

Supplemental Table 17: Metabolite levels for patients included in onset of fever analyses.

Patient	2HG	Acetic Acid	Buty-rate	Glyceric Acid	Keto-leucine	Lactate	Malate	Pentanoic Acid	Pro-pionate	Suc-cinate
0339.2013.001	123.6	1973.4	52.6	36.6	67.1	127.5	113.2	3.9	115.9	16.7
0339.2013.002	90.6	1334.6	5.8	61.4	19.1	14.7	207.3	0.4	211.2	744.4
0339.2013.003	82.6	1084.5	87.5	33.9	120.2	109.4	85.4	4.2	573.5	90.3
0339.2013.004	31.0	1948.0	100.7	34.5	145.7	13.8	92.1	22.5	808.4	125.6
0339.2013.005	63.2	1821.9	227.6	35.3	121.1	19.2	142.3	10.0	1329.9	600.9
0339.2013.006	43.4	784.0	35.9	12.3	11.0	3.2	14.5	20.7	499.2	56.5
0339.2013.007	138.7	1853.6	40.8	16.3	92.6	256.8	72.5	0.6	16.8	667.0
0339.2013.008	29.3	1315.1	93.2	22.5	96.6	5.5	38.4	23.1	414.3	94.6
0339.2013.026	37.8	2579.9	67.2	28.8	125.6	68.2	21.4	3.8	368.1	1265.9
0339.2013.027	53.7	1066.1	0.2	35.2	13.5	103.3	135.7	0.1	9.5	69.5
0339.2013.028	48.2	816.1	0.2	24.1	6.8	33.5	280.8	0.0	8.2	24.8
0339.2013.031	197.1	2450.4	12.1	21.2	48.2	14.1	99.7	1.0	162.5	724.5
0339.2013.033	18.7	1120.1	80.9	16.2	120.0	13.9	131.7	38.1	1138.2	121.3
0339.2013.034	2.0	430.9	22.5	3.4	9.3	3.3	15.5	7.3	123.6	11.0
0339.2013.036	149.8	4415.6	21.5	44.7	98.3	472.7	233.7	0.1	16.5	593.0
0339.2013.037	450.5	1424.2	76.1	47.3	131.2	524.3	117.2	22.9	2442.5	56.1
0339.2013.038	74.6	1319.9	2.1	21.9	61.4	17.9	128.7	0.3	350.3	30.0
0339.2013.039	138.3	1133.8	7.7	23.4	36.2	94.7	144.6	2.8	200.5	564.3
0339.2013.041	49.4	1384.8	35.0	36.4	18.7	35.9	68.4	2.3	31.6	366.9
0339.2013.043	30.5	1326.3	77.9	12.1	23.5	6.4	7.4	7.0	530.7	188.5
0339.2013.046	93.8	3007.6	61.5	50.4	84.2	652.9	284.9	3.2	104.5	585.4
0339.2013.050	14.2	2083.0	111.9	13.0	53.7	14.5	53.2	34.2	454.0	51.1
0339.2013.052	14.8	1635.0	0.8	20.5	6.6	145.1	74.7	0.4	9.8	41.2
0339.2013.055	12.7	1622.7	403.1	43.1	9.6	12.6	37.8	32.4	950.7	299.2
0339.2013.056	35.1	2660.6	0.1	30.6	13.8	181.8	176.9	0.2	7.5	21.4
0339.2013.057	27.9	1667.4	17.6	11.1	60.1	8.9	27.9	7.8	194.8	65.7
0339.2013.058	119.3	2598.7	7.5	22.7	71.5	210.6	119.7	0.2	25.9	874.0
0339.2013.059	155.9	2453.0	66.5	41.9	77.1	6.2	191.4	22.2	504.7	247.4
0339.2013.060	22.5	2077.7	184.8	30.4	40.7	7.3	98.4	43.0	699.0	50.8
0339.2013.062	11.3	1331.1	0.1	7.1	10.6	119.3	23.8	0.7	2.9	16.1
0339.2013.063	121.4	2325.8	19.2	23.1	15.4	66.0	22.4	0.7	218.5	216.0
0339.2013.065	84.7	2436.3	3.0	42.8	39.7	158.3	222.7	0.8	72.9	1693.9
0339.2013.066	172.5	4507.2	28.0	32.4	37.0	339.0	76.3	15.7	336.2	92.4
0339.2013.067	39.1	2007.9	263.1	30.8	23.4	68.7	28.3	39.5	889.0	87.6
0339.2013.075	181.9	3667.1	61.0	37.9	141.4	89.3	112.5	25.9	94.0	1039.3
0339.2013.076	56.5	1709.1	169.6	56.8	166.9	37.2	219.5	46.1	771.6	202.6
0339.2013.077	2326.1	68.6	0.0	0.0	0.0	1459.1	2444.3	9.1	0.0	18046.6
0339.2013.078	184.3	3611.7	0.3	55.4	131.8	395.9	141.5	0.3	84.9	2049.9
0339.2013.081	31.5	4201.5	264.1	82.7	37.9	1548.1	214.5	9.1	899.8	2282.6

Continued: Supplemental Table 17

Patient	2HG	Acetic Acid	Buty-rate	Glyceric Acid	Keto-leucine	Lactate	Malate	Pentanoic Acid	Pro-pionate	Suc-cinate
0339.2013.085	61.5	2350.7	68.0	24.1	132.0	17.0	20.3	25.7	682.3	126.9
0339.2013.087	442.2	7288.6	0.1	63.6	14.3	1219.5	554.2	0.1	14.2	893.1
0339.2013.089	324.7	3338.6	189.8	67.5	196.0	2373.7	217.4	15.1	424.8	4087.1
0339.2013.091	117.7	1567.6	0.5	55.9	16.9	361.4	227.1	0.2	20.2	1149.5
0339.2013.092	22.4	961.8	71.2	44.7	62.5	22.4	68.9	15.5	257.7	134.1
0339.2013.094	66.5	1110.4	0.3	75.5	9.4	31.1	183.5	0.1	7.6	1157.4
0339.2013.098	33.6	2577.9	1.6	23.1	40.5	390.2	49.2	0.4	44.9	328.7
0339.2013.099	459.3	2779.3	6.9	79.7	55.5	37.6	341.5	0.6	223.9	1709.2
0339.2013.100	63.9	1790.9	0.3	38.1	2.8	64.4	201.4	0.1	12.3	462.5
15-0780-002	8.6	1417.4	87.3	20.2	55.7	8.1	53.4	27.9	430.4	755.9
15-0780-004	10.5	1871.8	61.3	28.3	19.8	5.6	92.9	17.4	207.6	409.9
15-0780-006	68.1	2792.3	4.8	31.6	19.5	8.3	58.5	0.8	496.5	27.4
15-0780-008	143.8	3370.3	10.1	23.3	97.3	45.4	80.4	0.3	26.1	933.7
15-0780-013	21.1	2218.9	0.2	26.0	14.2	247.8	130.3	0.1	12.2	81.2
15-0780-016	53.0	2121.9	0.7	37.5	11.9	338.6	772.0	0.1	6.3	607.1
15-0780-020	7.5	2580.2	0.3	111.7	17.2	211.9	195.6	0.1	9.7	34.6
15-0780-025	126.4	3002.7	87.1	55.4	87.8	16.1	29.0	15.7	265.8	169.3
15-0780-026	37.5	2216.8	120.3	49.4	66.3	11.6	154.2	30.3	1237.6	66.6
15-0780-029	7.1	1423.0	0.1	26.5	6.0	169.0	95.3	0.2	4.8	22.7
15-0780-030	5.0	1149.5	228.2	19.0	20.0	7.2	15.0	45.6	699.5	36.8
15-0780-033	330.1	3070.7	69.1	57.4	178.0	14.4	189.3	49.5	684.0	152.0
15-0780-034	14.4	2338.5	69.4	31.0	56.0	8.6	76.7	3.4	898.3	373.4
15-0780-035	222.5	2468.9	29.2	64.6	186.5	459.1	170.9	2.4	1941.0	583.9
15-0780-040	75.1	3873.6	64.4	44.7	232.2	21.6	45.4	7.7	206.3	1920.1
15-0780-041	3.1	666.2	39.2	10.4	10.8	2.2	54.3	17.5	366.3	125.7
15-0780-043	79.2	677.6	0.3	13.2	3.6	25.8	146.7	0.1	10.5	496.8
15-0780-044	22.5	1744.9	245.0	34.4	13.4	9.1	101.1	24.9	815.3	304.6
15-0780-046	23.0	1182.5	57.4	32.0	114.3	197.4	143.7	1.4	379.9	1013.5
15-0780-052	0.5	144.6	0.0	2.0	1.0	30.4	2.1	0.1	0.6	3.1
15-0780-055	65.0	1795.3	9.5	39.3	9.0	19.8	81.9	0.7	367.4	271.9
15-0780-056	404.5	1977.0	41.5	29.4	15.5	56.1	124.4	2.0	1085.4	552.7
15-0780-058	2.3	539.9	57.0	5.1	0.7	5.2	8.9	9.1	107.8	227.4
15-0780-059	17.0	898.4	52.6	9.7	1.3	4.6	8.5	31.5	236.0	502.9
15-0780-060	39.3	1244.5	261.9	23.8	5.3	33.0	82.3	48.2	1516.6	1025.4
15-0780-063	12.2	2413.0	102.2	24.8	98.7	7.5	21.6	3.5	616.0	170.5
